# Low-dose allergen targeting to cDC2 induces long-term protection associated with induction of regulatory-like IL-10^+^ Foxp3^-^ CD8^+^ T cells in mice

**DOI:** 10.3389/fimmu.2026.1846923

**Published:** 2026-08-25

**Authors:** Arpa Aintablian, Laura Cyran, Moutaz Helal, Christian H.K. Lehmann, Fabian Imdahl, Tom Gräfenhan, Angela Riedel, Diana Dudziak, Andreas Kerstan, Manfred B. Lutz

**Affiliations:** 1 Institute for Virology and Immunobiology, University of Würzburg, Würzburg, Germany; 2 Mildred Scheel Early Career Centre (MSNZ) for Cancer Research, Würzburg, University Hospital Würzburg, and University of Würzburg, Würzburg, Germany; 3 Department of Dermatology, Venerology and Allergology, University Hospital Würzburg, Würzburg, Germany; 4 CELLIKON, Department of Dermatology, University Hospital Erlangen, Erlangen, Germany; 5 Deutsches Zentrum Immuntherapie (DZI), Erlangen, Germany; 6 Department of Pediatrics and Adolescent Medicine, Friedrich-Alexander-University Erlangen-Nuremberg, Erlangen, Germany; 7 Friedrich-Alexander-University Erlangen-Nuremberg (FAU) Profile Center Immunomedicine (FAU I-MED), Erlangen, Germany; 8 Single-Cell Center Würzburg, Helmholtz Institute for RNA-based Infection Research, Würzburg, Germany; 9 Core Unit Systems Medicine, University of Würzburg, Würzburg, Germany; 10 Laboratory of Dendritic Cell Biology, Department of Dermatology, University Hospital Erlangen, Erlangen, Germany; 11 Institute of Immunology, University Hospital Jena, Friedrich Schiller University, Jena, Germany; 12 Cluster of Excellence Balance of the Microverse, Friedrich Schiller University Jena, Jena, Germany

**Keywords:** allergy, CD8 regulatory T cells, dendritic cells, targeting, therapy

## Abstract

The prevalence of allergic diseases is rising worldwide. Desensitizing immunotherapy is currently the only available treatment, but it is often ineffective for certain allergies, underscoring the need for new treatments. This study tested in an Ovalbumin (OVA)-mediated allergy mouse model whether direct allergen targeting to DC subsets can induce desensitization. A Standard desensitization protocol was compared with an antibody-OVA fusion protein targeting the conventional cDC1 or cDC2 subsets via their respective receptors CD205 (CD205-Fp) or DCIR2 (DCIR2-Fp). Allergen targeting to cDC2 provided long-lasting protection for up to 56 days after desensitization with only nanogram doses of allergen, whereas the Standard therapy protected for only 20 days, requiring microgram doses and repeated injections with Alum adjuvant. CD205-Fp showed no protective effect. Blocking IL-10 either before administering DCIR2-Fp or before allergen challenge completely abolished protection. Standard therapy had moderate effects on CD4^+^ T cells, with early expansion of IL-10^+^ Foxp3^-^ T regulatory type-1 cells (Tr1), followed by an increase in both IL-10^+^ and IL-10^-^ Foxp3^+^ regulatory T cells (Foxp3^+^ Treg). DCIR2-Fp therapy initially expanded IL-10^+^ and IL-10^-^ Foxp3^+^ Treg, but these levels returned to baseline. On day 80, we identified Foxp3^-^ CD44^high^ Treg-like CD8^+^ as the primary source of IL-10. Overall, our data suggest that cDC2 allergen targeting in allergic mice offers a potent, long-lasting IL-10-dependent desensitizing effect.

## Introduction

The global prevalence of allergic diseases continues to rise, with IgE-mediated type I allergies, including hay fever, food allergies, and allergic asthma, escalating rapidly (1–4). Allergen Immunotherapy (AIT) is the only curative treatment that can induce long-term desensitization to allergens; however, success rates vary depending on the type of allergy. AIT with Apidae (bees) and Vespidae (wasps) venom is a prime example of highly effective immunological tolerance induction, protecting up to 95% of affected individuals from recurrent anaphylactic reactions following a wasp sting (5–7). Clinical case series demonstrate that this protection against anaphylaxis develops early, within a few days, during the induction or dose-escalation phase, leading to short-term tolerance that appears to depend on Tr1. CD4^+^ Tr1 are characterized by their lack of Foxp3 expression and high IL-10 production, as well as variable levels of other immunoregulatory receptors such as LAG3, PD-1, CTLA-4, TIM-3, TIGIT, and CD39 (8–11). They can develop from naive CD4^+^ T cells, likely passing through a stage of T cell anergy (12), or from differentiated T helper cell subsets (13).

In contrast, a network of various regulatory immune cell types and mechanisms contributes to the long-term success of AIT (14–16), with T cells still playing a central role. Among those, Foxp3^+^ Treg are key mediators of adaptive immune tolerance that suppress excessive immune responses, including allergic reactions (17, 18), and have been shown to persist long term to sustain tolerance, as demonstrated in patients with wasp venom allergy (19). Subtypes include CD4^+^ Foxp3^+^ Treg, which are either generated in the thymus (tTreg) or converted from peripheral naive T cells (pTreg) in lymph nodes (LN) (20). More recently, Foxp3^+^ CD8^+^ Treg have also been found to represent essential immune regulators, but they appear to exhibit a heterogeneous marker profile, and an additional Foxp3^-^ CD8^+^ Treg subset remains poorly defined (21). Upon activation, both CD4^+^ and CD8^+^ Foxp3^+^ Treg can produce IL-10, among other immunosuppressive mechanisms (22). The roles of Foxp3^+^ CD4^+^ and Foxp3^+^ CD8^+^ Treg and Tr1 in allergy and AIT are not yet fully understood, especially the potential role of long-lived IL-10^+^ Foxp3^+^ Treg or Tr1 (23), and which DC subset is responsible for their induction and maintenance.

Both conventional DC (cDC1 and cDC2) subsets are known to help maintain tolerance to self-antigens and harmless foreign antigens (24, 25). Lung DC have been shown to take up OVA allergen and transport it to the draining LN, where OVA-specific Tr1 are induced (26, 27). The maturation state of these CD205^+^ DC, characterized by increased MHC II and costimulatory molecules along with IL-10 secretion, resembles that of steady-state migratory cDC1. The latter are well documented as tolerogenic, picking up self-antigens in peripheral tissues, undergoing partial maturation, migrating into the T cell areas of the draining LN, and inducing Tr1 or Foxp3^+^ Treg, depending on their immunosuppressive cytokine secretion pattern (28–32). All DC subsets are shown to undergo semi-maturation, migrate, take up soluble antigens within the LN from the reticular conduit system, and induce tolerance in their immature DC state (24, 32, 33). Although the role of DC in the induction and regulation of Tr1 and Foxp3^+^ Treg is well established, their role in allergy desensitization remains understudied.

For desensitization success after AIT, DC play a crucial role in promoting various regulatory T cell subsets, and are therefore broadly referred to as regulatory DC, without specifying a particular cDC subset. Since cDC1 and cDC2 differ in their ability to induce effector T cell responses (34), this dichotomy may also apply to tolerance induction. Therefore, selectively targeting allergens to cDC1 or cDC2 could help clarify their individual roles during desensitization. The surface molecules CD205 and DCIR2, expressed on cDC1 and cDC2, respectively, have been successfully used for antigen-specific targeting in mice using fusion proteins, in which OVA or other antigens were conjugated to murine antibodies recognizing CD205 (CD205-Fp) or DCIR2 (DCIR2-Fp) (35). These fusion proteins have been applied in several murine disease models (36, 37). Targeting cDC1 via CD205 has been shown to induce Foxp3^+^ Treg (25), and this approach has been successfully applied in the treatment of murine autoimmune diseases, such as experimental autoimmune encephalomyelitis (EAE) and type 1 diabetes in NOD mice (38, 39), and in human skin explants (40). To date, targeting cDC2 via DCIR2 in mice has been used in various models of melanoma treatment, for antigen uptake and presentation to study T cell responses (37, 41, 42). Targeting of cDC1 or cDC2 subsets by allergens for desensitization has not yet been explored.

Here, we used CD205-Fp and DCIR2-Fp as targeted treatments to selectively engage cDC1 and cDC2, respectively, in a well-established OVA-mediated type I allergy mouse model. The DCIR2-Fp therapy was compared with the Standard therapy for long-lasting desensitization. Our results revealed that targeting cDC2 was as effective as the Standard therapy while requiring lower allergen doses and suggested a role for IL-10-producing Foxp3^-^ CD44^high^ Treg-like CD8^+^ as key mediators of allergen desensitization.

## Results

### Both the DCIR2-Fp and Standard therapy provide equivalent short-term protection against allergen challenge

**Figure 1:**
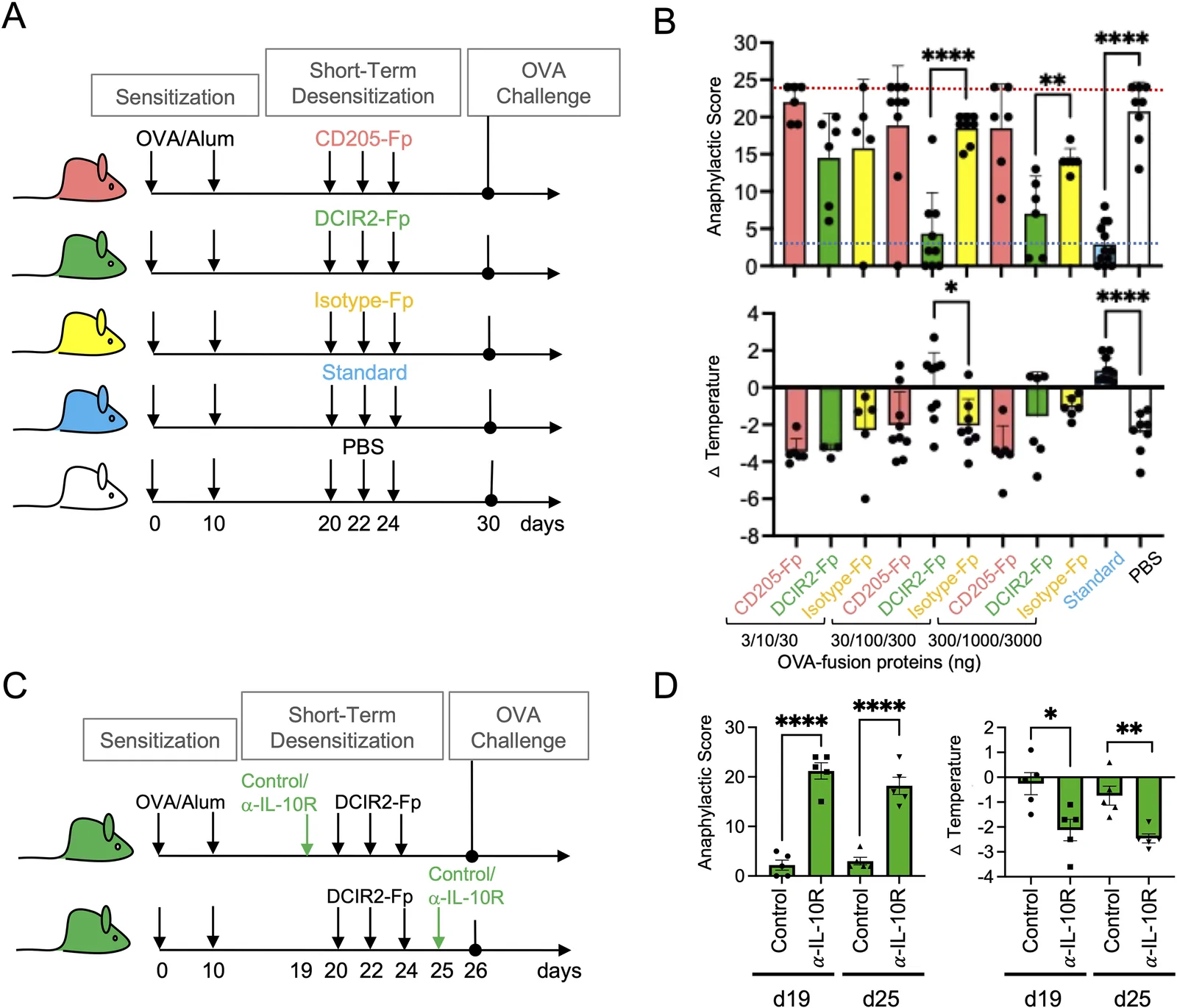
DCIR2-Fp and Standard therapy protect mice from anaphylactic shock. **(A)** Mice were sensitized at days 0 and 10 by i.p. injections of 10 µg OVA/Alum. Short-term densitization was followed on days 20, 22, and 24 by three i.p. injections of either PBS alone, OVA in PBS (Standard), or the indicated antibody-OVA fusion proteins at one of three dose levels (low, intermediate, or high). On day 30, mice were challenged by an i.p. injection of a high dose of 40 µg OVA in PBS. **(B)** Anaphylactic scores and changes in body temperature were assessed following allergen challenge as shown in **(A)** Temperature change was calculated as the difference between pre-challenge and 45-min post-challenge measurements. The red and blue dotted lines indicate the highest and lowest observed anaphylactic scores, respectively. Data are from *n* = 5–10 mice per group. **(C)** An anti-IL-10R blocking antibody or an isotype control antibody was administered either before (day 19) or after (day 25) desensitization with an intermediate dose of DCIR2-Fp. Allergen challenge was performed on day 26. **(D)** Anaphylactic scores and changes in body temperature as shown in C were assessed following allergen challenge. Temperature change was calculated as the difference between pre-challenge and 45-min post-challenge measurements. Data are from *n* = 5 mice per group. Statistical analyses were performed by unpaired, two-tailed *t*-tests specified *a priori* between groups and their respective control were applied and are indicated as ****p < 0.0001; **p < 0.01; *p < 0.05.

Since AIT is an approved treatment for allergies in humans, we translated it to mice and used it as our reference Standard therapy to compare it to the efficacy of two OVA fusion protein-based therapies in mice. The latter included CD205-Fp and DCIR2-Fp, which were designed to selectively target cDC1 and cDC2, respectively. To assess the effectiveness of DC targeting versus non-targeted OVA/antibody application, we used an isotype control antibody conjugated to OVA. Allergen-sensitized mice received different therapy regimens, including titrated amounts of fusion proteins, Standard OVA/PBS, or only PBS injections i.p. on days 20, 22, and 24. An OVA allergen challenge was performed on day 30, and the allergen score was determined (**Figure 1A**).

We found that OVA-sensitized mice receiving only PBS showed a high anaphylactic score and a drop in rectal temperature after allergen challenge, indicating a persistent allergic response. Mice that received an intermediate (30/100/300 ng) or a high dose (300/1000/3000 ng) of DCIR2-Fp, but not a low dose (3/10/30 ng), showed a reduced anaphylactic score and an increase in body temperature, like the group that received short-term Standard OVA therapy. All doses of CD205-Fp or Isotype-Fp injections did not affect anaphylaxis and body temperature, and these groups appeared similar to the PBS group (**Figure 1B**). These findings suggest that the intermediate-dose regimen with DCIR2-Fp was sufficient to provide full protection against anaphylaxis, comparable to OVA Standard therapy, while requiring substantially lower allergen doses. Hence, the intermediate dose regimen for DCIR2-Fp was selected for further experiments.

To determine whether IL-10 contributes to successful DCIR2-Fp-induced desensitization and protection against allergen challenge, an anti-IL-10 receptor (anti-IL-10R) antibody or a control antibody was injected either directly before (on day 19) or after (on day 25) the intermediate dose regimen of DCIR2-Fp desensitization (days 20, 22, and 24). An allergen challenge was performed on day 26 to test whether immediate IL-10 production was responsible for protection (**Figure 1C**). Blocking IL-10 before or after desensitization markedly increased the anaphylactic score and significantly lowered rectal temperature as compared to control antibody injection (**Figure 1D**). This indicates that IL-10 is crucial for short-term desensitization by DCIR2-Fp, as indicated by the protection against OVA challenge.

### OVA standard therapy and DCIR2-Fp promote the induction of Tr1 and Treg, respectively

**Figure 2:**
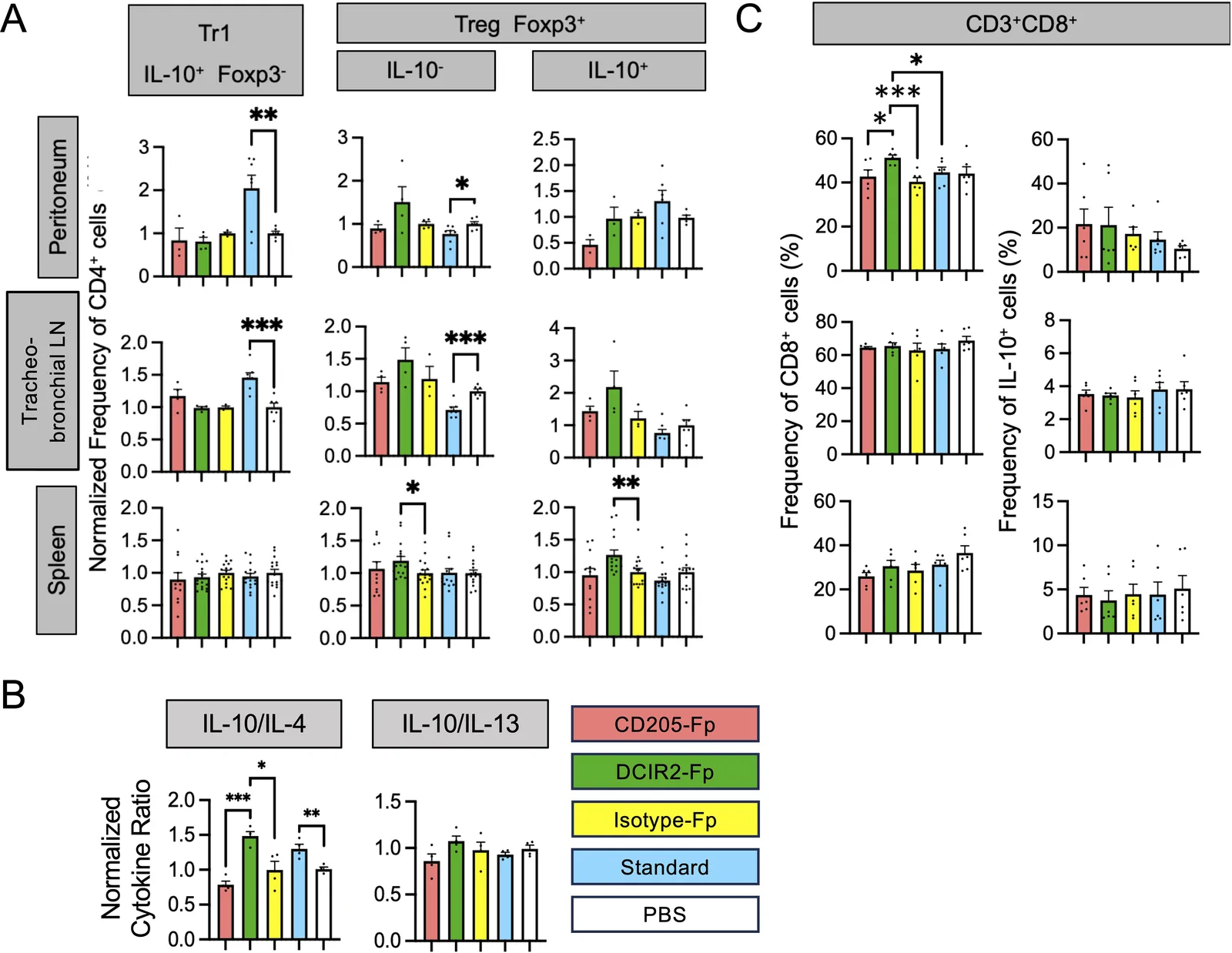
Short-term standard therapy promotes local Tr1 while DCIR2-Fp induces Foxp3^+^ Treg in the spleen. Sensitization and desensitization of mice were performed as outlined in **Figure 1A**. **(A)** On day 30, cells from the peritoneal cavity, tracheobronchial lymph nodes, and spleen were stimulated with PMA, ionomycin, and brefeldin A for 5 h and subsequently analyzed by flow cytometry. Graphs show the normalized frequencies of cells within the Tr1 and IL-10^-^/^+^ Foxp3^+^ Treg gates under the indicated treatment conditions and in the indicated organs. The gating strategy is shown in **Supplementary Figure 1A**. Data are from *n* = 3–6 mice per group. Additional technical replicates were included for spleen samples. Normalization was calculated by setting the PBS group = 1 and relating it to the experimental groups (group x 1/PBS). **(B)** On day 30, splenocytes were stimulated with PMA and ionomycin for 72 h, to induce cytokine production to detectable levels. Cytokine concentrations in the culture supernatants were quantified using a LegendPlex assay. Graphs show the ratios of IL-10 to IL-4 and IL-10 to IL-13 under the indicated treatment conditions. Cytokine concentrations were normalized to the respective control group prior to ratio calculation. Normalization was calculated by setting the PBS group = 1 and relating it to the experimental groups (group x 1/PBS). Data are from *n* = 4 mice per group. **(C)** Graphs show the frequencies of CD8^+^ T cells and the frequencies of IL-10^+^ cells among CD8^+^ T cells under the indicated treatment conditions and in the indicated organs. The gating strategy is shown in **Suppl. Figure 1A**. Data are from *n* = 6 mice per group. **(A)**, **(C)** Values from CD205-Fp and DCIR2-Fp groups were normalized to the corresponding Isotype-Fp control group. Values from the Standard therapy group were normalized to the PBS control group. Statistical analyses were performed by ANOVA to evaluate overall differences but did not appear to be significant. In addition, unpaired, two-tailed *t*-tests specified *a priori* between groups and their respective control were applied and are indicated as ***p < 0.001; **p < 0.01; *p < 0.05.

Given that cDC2 targeting would lead to antigen presentation of the OVA allergen to T cells, we aimed to identify the specific T cell subtypes that produce IL-10, as well as IL-4 and IL-13, as hallmarks of T helper 2 cells (Th2). Therefore, mice were sensitized and desensitized as shown in **Figure 1A**. On day 30, single-cell suspensions from spleen, peritoneum, and tracheobronchial LN were stimulated *in vitro* with PMA/ionomycin in the presence of brefeldin A and analyzed by flow cytometry. For the detection of IL-10, highly sensitive IL-10-β-lactamase reporter (ITIB) reporter mice were used (43). CD3^+^ CD4^+^ CD44^high^ cells were divided into IL-10^+^ and IL-10^-^ Foxp3^+^ Treg, IL-10^+^ Foxp3^-^ Tr1 and IL-10^-^ Foxp3^-^ T cells (**Suppl. Figure 1A**). Following Standard therapy, a significant increase in IL-10^+^ Tr1 and a simultaneous decrease in IL-10^-^ Foxp3^+^ Treg were observed both in the peritoneum and tracheobronchial LN, but not in the spleen (**Figure 2A**). In contrast, DCIR2-Fp therapy caused an increase in IL-10^+^ and IL-10^-^ Foxp3^+^ Treg in the spleen and showed a trend toward increased IL-10^-^ Foxp3^+^ Treg in the peritoneum and IL-10^+^ and IL-10^-^ Foxp3^+^ Treg in the tracheobronchial LN (**Figures 2A**; **Supplementary Figure 2A**). These findings suggest that the Standard therapy provides local protection against anaphylaxis via Tr1. At the same time, DCIR2-Fp-therapy generates systemic Foxp3^+^ Treg cells that may protect against allergic shock.

To gain a broader understanding of cytokine production, splenocytes were stimulated for 3 days with PMA/ionomycin, and the cytokines in the supernatant were measured using a LegendPlex™ assay. We observed a tendency toward higher IL-10 production in mice that received Standard desensitization, although there were no significant differences in the production of IL-10, IL-4, and IL-13 between the different treatment groups (**Suppl. Figure 2B**). However, both the Standard and DCIR2-Fp therapy groups exhibited significantly higher IL-10/IL-4 ratios, with the DCIR2-Fp group also showing a trend toward an increased IL-10/IL-13 ratio (**Figure 2B**). Thus, the increased IL-10/IL-4 ratio is consistent with a relative shift toward lower IL-4 and IL-13, compared with IL-10, rather than a significant increase in absolute IL-10 production.

To further characterize the phenotype of the regulatory T cell populations after successful short-term desensitization, the expression of well-described candidate activation and exhaustion markers (12, 44–46) was analyzed by flow cytometry in the spleen (example stainings in **Supplementary Figure 1B**). An upregulation of TIGIT was observed on Tr1 in mice receiving Standard therapy (**Supplementary Figure 2C**). DCIR2-Fp therapy showed a significant increase in the expression of TIGIT and FR4 on IL-10^+^ Foxp3^+^ Treg. At the same time, the expression of LAG3 and CTLA-4 remained unchanged across all groups (**Supplementary Figure 2C**). Analysis of the CD8^+^ T cell compartment showed an increased frequency of total CD8^+^ T cells in the peritoneum of the DCIR2-Fp group. However, these cells did not exhibit increased IL-10 production (**Figure 2C**), and no changes were detected in the tracheobronchial LNs, spleen, or the expression of Foxp3, CTLA-4, TIGIT, or LAG3 (**Supplementary Figure 2D**).

Together, at this early time point (day 30) after desensitization, elevated frequencies of IL-10^+^ T cells could be detected only for Tr1 after Standard therapy and for Foxp3^+^ Treg after DCIR2-Fp treatment.

### DCIR2-Fp desensitization provides IL-10-dependent long-term protection

**Figure 3:**
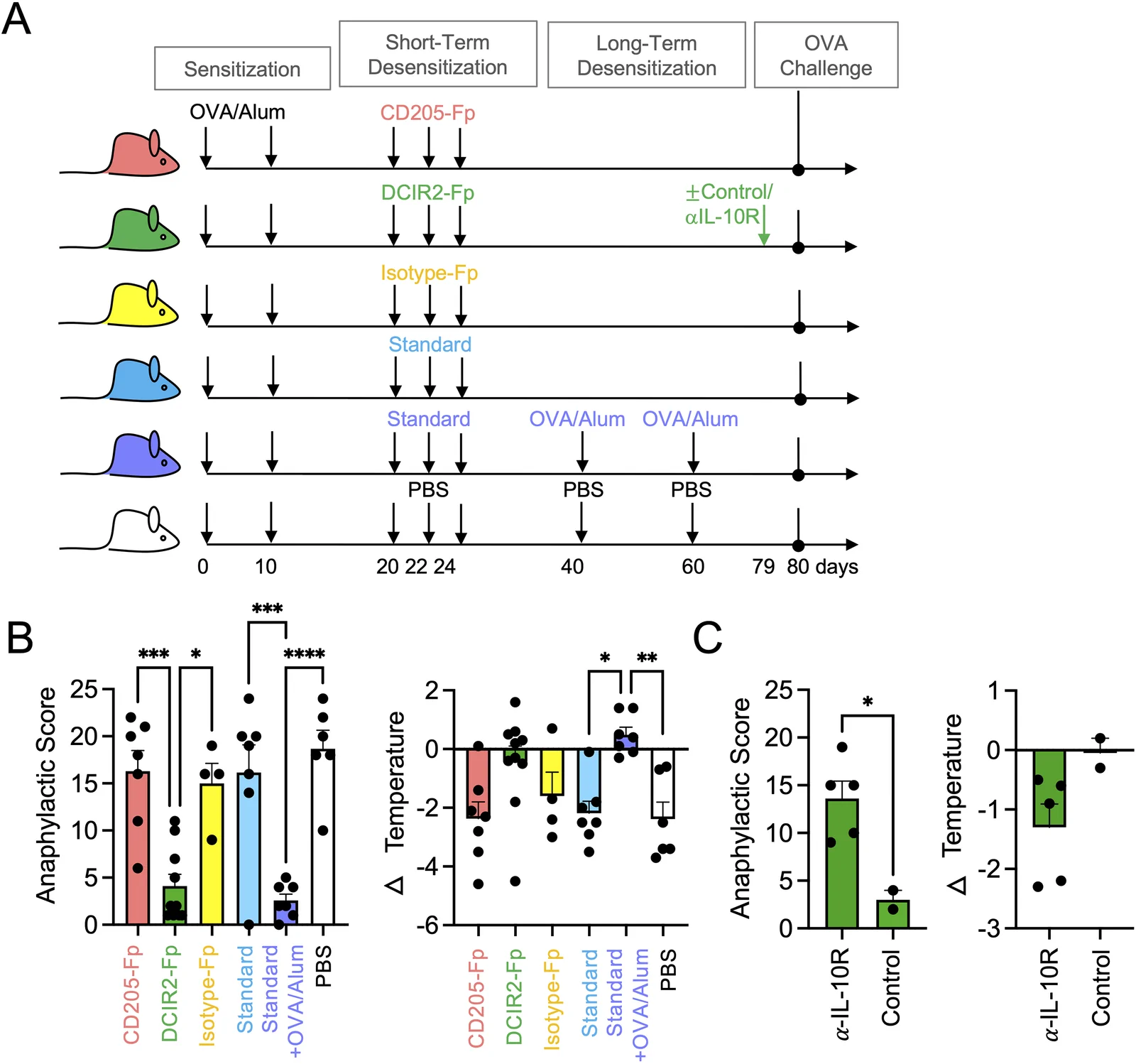
Standard therapy requires additional OVA/Alum to induce long-term protection, while DCIR2-Fp desensitization induces durable protection without long-term desensitization, in an IL-10-dependent manner. **(A)** Mice were sensitized at days 0 and 10 by i.p. injections of 10 µg OVA/Alum. Desensitization was performed on days 20, 22, and 24 by i.p. injections of PBS alone, OVA in PBS (Standard), or intermediate doses (30, 100, and 300 ng) of the indicated antibody-OVA fusion proteins. Mice in the Standard therapy group additionally received OVA/Alum injections on days 40 and 60 (long-term desensitization). On day 80, all groups were challenged i.p. with a high dose (40 µg) of OVA in PBS. **(B)** Anaphylactic scores and changes in body temperature were assessed following allergen challenge. Temperature change was calculated as the difference between pre-challenge and 45-min post-challenge measurements. Data are from *n* = 4–9 mice per group. **(C)** On day 79, mice desensitized with DCIR2-Fp received either an anti-IL-10R blocking antibody or an isotype control antibody. Allergen challenge was performed on day 80 by i.p. injection of a high dose of 40 µg OVA in PBS. Anaphylactic scores and changes in body temperature were assessed following allergen challenge. Temperature change was calculated as the difference between pre-challenge and 45-min post-challenge measurements. Data are from *n* = 2–5 mice per group. Statistical analyses were performed by ANOVA to evaluate overall differences but did not appear to be significant. In addition, unpaired, two-tailed *t*-tests specified *a priori* between groups and their respective control were applied and are indicated as ****p < 0.0001; ***p < 0.001; **p < 0.01; *p < 0.05.

For long-term efficacy, maintenance therapy is essential in human AIT. Therefore, we applied long-term Standard therapy by administering OVA with Alum again on days 40 and 60 as long-term desensitization, reflecting the maintenance phase of clinical treatment (7). We then compared this long-term Standard therapy regimen with the DCIR2-Fp and CD205-Fp therapies, in which no further long-term desensitization injections were administered after day 24 (**Figure 3A**). Mice desensitized with DCIR2-Fp, but not with CD205-Fp or Isotype-Fp, showed low anaphylactic scores and higher rectal temperatures following OVA challenge on day 80 (i.e. 56 days after desensitization), indicating long-term protection from anaphylaxis after only three injections for desensitization. Mice that received long-term Standard desensitization were also protected on day 80, as expected, but required two additional OVA/Alum treatments since protection on day 80 was not achieved with the short-term Standard therapy alone (**Figure 3B**).

To determine whether the long-term protection induced by DCIR2-Fp treatment is also IL-10-dependent, mice were given an anti-IL-10R blocking antibody 24 h before allergen challenge (**Figure 3A**). Blocking the IL-10 receptor significantly increased the anaphylactic scores and lowered rectal temperatures in the mice compared to those that received a control antibody (**Figure 3C**). In conclusion, both short- and long-term protection offered by DCIR2-Fp therapy depends on IL-10.

### Long-term standard therapy promotes Foxp3^+^ Treg cells, whereas both desensitizing therapies increase the splenic IL-10/IL-4 ratio on day 80

**Figure 4:**
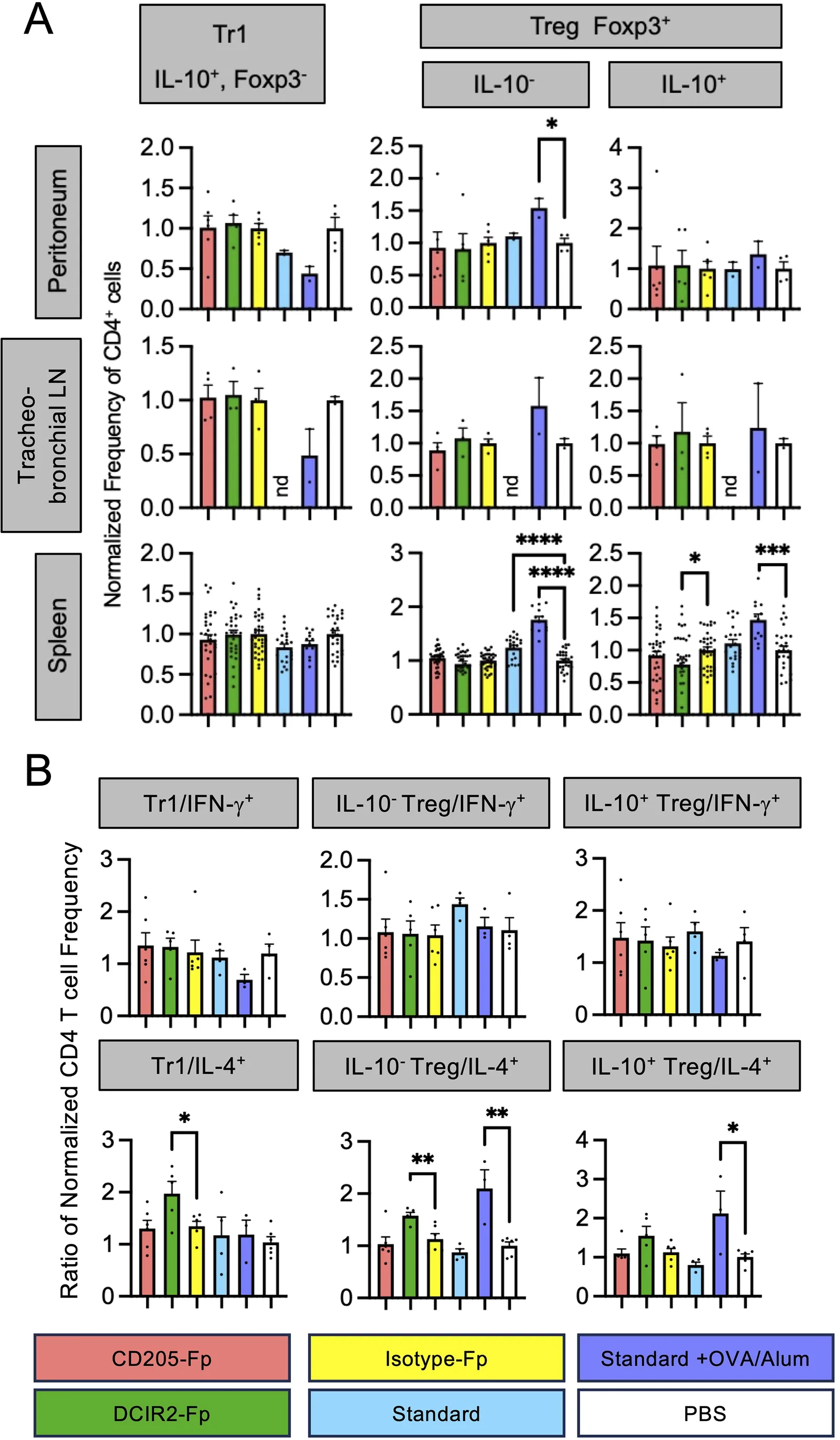
Only long–term Standard therapy induces Foxp3^+^ Treg, but both desensitizing therapies increase IL-10/IL-4 ratios at day 80 in the spleen. Sensitization and desensitization were performed as outlined in **Figure 3A**. **(A)** On day 80, cells from the peritoneal cavity, tracheobronchial LN, and spleen were stimulated with PMA/ionomycin, and brefeldin A for 5 h and subsequently analyzed by flow cytometry. The gating strategy is shown in **Suppl. Figure 1A**. Graphs show the normalized frequencies of cells within the Tr1 and IL-10^-^/^+^ Treg gates under the indicated treatment conditions and in the indicated organs. Data are from *n* = 3–7 mice per group. Additional technical replicates were included for the spleen. Normalization was calculated by setting the PBS group = 1 and relating it to the experimental groups (group x 1/PBS). **(B)** Graphs show the ratios of normalized frequencies of cells within the Tr1 and IL-10^-^/^+^ Foxp3^+^ Treg gates to IFN-γ^+^ or IL-4^+^ T cells in the spleen under the indicated treatment conditions. Data are from *n* = 3–6 mice per group. Values from the CD205-Fp and DCIR2-Fp groups were normalized to the corresponding Isotype-Fp control group. Normalization was calculated by setting the PBS group = 1 and relating it to the experimental groups (group x 1/PBS). Values from the Standard therapy groups were normalized to the PBS control group. Statistical analyses were performed by ANOVA to evaluate overall differences but did not appear to be significant. In addition, unpaired, two-tailed *t*-tests specified *a priori* between groups and their respective control were applied and are indicated as ****p < 0.0001; ***p < 0.001; **p < 0.01; *p < 0.05.

Since two subsets of regulatory T cells (Tr1 and Foxp3^+^ Treg) have been reported to contribute to AIT-mediated protection (19), we analyzed their frequencies in different organs also in our long-term therapy settings. Long-term Standard therapy induced IL-10^-^ Foxp3^+^ Treg in the peritoneum, tracheobronchial LN, and systemically in the spleen (**Figures 4A**; **Supplementary Figure 3A**). A significant increase in the frequency of IL-10^+^ Foxp3^+^ Treg was also observed in the spleen (**Figure 4A**). Surprisingly, no significant differences were found in the frequencies of Tr1 and Foxp3^+^ Treg with DCIR2-Fp therapy compared to the control group across all examined organs (**Figure 4A**).

We next analyzed the ratios of intracellular cytokine-producing T cell subsets. Long-term Standard therapy resulted in increased IL-10^-^ Foxp3^+^ Treg/IL-4^+^ and IL-10^+^ Foxp3^+^ Treg/IL-4^+^ ratios in the spleen, consistent with their elevated frequencies. In contrast, Tr1/IL-4^+^ and Tr1- or Foxp3^+^ Treg/IFN-γ ratios remained unchanged (**Figure 4B**). DCIR2-Fp treatment also increased the IL-10^-^ Foxp3^+^ Treg/IL-4^+^ ratio and, in addition, elevated the Tr1/IL-4^+^ ratio, an effect not observed with long-term Standard therapy (**Figure 4B**). Together, no increase of IL-10 producing CD4^+^ T cell subsets could be observed but an elevated IL-10/IL-4 ratio, suggesting reduced IL-4^+^ Th2 cells, similar as seen after short-term therapy.

When analyzing activation or exhaustion markers on Foxp3^+^ Treg and Tr1 spleen cells, mice that received long-term Standard therapy showed significantly higher expression of TIGIT, FR4, CTLA-4, CD69, CD73, and a trend toward increased LAG3 expression within the IL-10^-^ Foxp3^+^ Treg (**Supplementary Figure 3B**). In contrast, IL-10^+^ Foxp3^+^ Treg subset remained largely unaffected, with only CD69 being significantly increased (**Supplementary Figure 3B**). LAG3 was the only marker significantly upregulated by Standard therapy on Tr1. (**Supplementary Figure 3B**). The mice treated with DCIR2-Fp did not show increased expression of any of these markers in either T cell subset (**Supplementary Figure 3B**). These data suggest that sources other than CD4^+^ T cells produce the IL-10 that contributes to their long-term protection.

### scRNA-seq reveals Treg-like CD8^+^ cells as the principal IL-10-producing cells in the spleen

**Figure 5:**
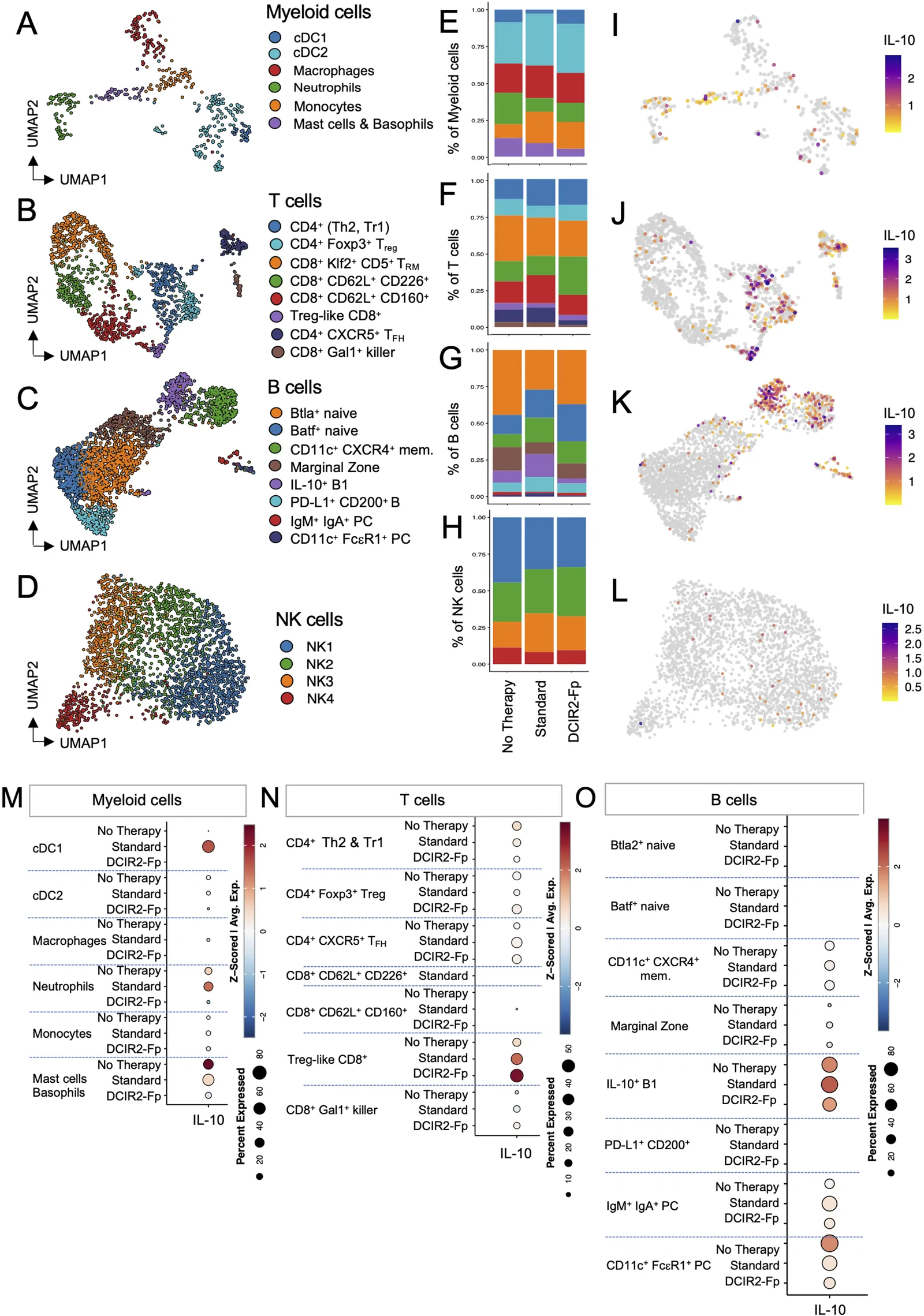
Identification of IL-10-producing cells by scRNA-seq in spleen at day 80. Mice were sensitized and desensitized as outlined in **Figure 3A**. and challenged at day 80 i.p. with 40µg OVA in PBS. Spleen cells of all groups were collected and enriched for otherwise scarce myeloid cells by sorting CD45^+^ CD11b^+^ cells and then pooling them with the total spleen cells at a 1:1 ratio. Then the pooled cells were subjected to scRNA-seq. **(A–D)** UMAP plots of scRNA-seq data showing annotated major cell populations, including myeloid cells, T cells, B cells, and NK cells. For cell annotation, data were pooled from mice receiving no therapy, long-term Standard therapy, or DCIR2-Fp therapy. **(E–H).** Stacked bar plots showing the relative proportions of cell types shown in A–D across the indicated treatment conditions. **(I–L).** UMAP plots showing normalized expression of *Il10* across the indicated cell populations. **(M–O).** Dot plots showing Z-scores of average *Il10* expression across the indicated cell types and treatment conditions.

To identify splenic cell types producing IL-10 other than CD4^+^ Foxp3^+^ Treg and Tr1 populations, scRNA-seq was performed on splenocytes collected on day 80 from mice that received long-term Standard therapy or DCIR2-Fp therapy and were compared to samples obtained from untreated allergic animals. Before sorting, cells were stimulated *in vitro* for 3 h with PMA/ionomycin to enhance transcriptional activity. To prevent underrepresentation of the less frequent myeloid cells, spleen cells were sorted into CD45^+^ CD11b^+^ and CD45^+^ CD11b^-^ fractions and pooled at a 1:1 ratio before processing for scRNA seq. Myeloid cells, T cells, B cells, and NK cells were identified and annotated based on characteristic markers (**Figures 5A–H**; **Supplementary Figures 4A–D**). UMAP plots showing IL-10 expression across all experimental conditions enabled the identification of clusters of IL-10-producing cells (**Figures 5I–L**). NK cells did not contribute to IL-10 production, and myeloid cells only moderately upregulated IL-10 transcripts within neutrophil and mast cell/basophil clusters. Strong IL-10 producers were found in clusters of T cells and B cells (**Figures 5I–L**).

Selected genes in the T cell clusters further supported the subset annotation of IL-10-producing T cells (**Supplementary Figure 5**). Within the CD4^+^ cluster, Gata3^+^ cells could be further divided into those polarized more toward Th2 (IL-10^low^ IL-4^+^ IL-13^+^) and others resembling a Tr1 profile (IL-10^high^ IL-4^-^ IL-13^-^), as well as a distinct population of CD4^+^ T_FR_ cells (CXCR5^+^ IL-10^+^ Foxp3^+/-^ CTLA-4^+^). Unexpectedly, a population of CD8^+^ T cells was identified that expressed transcripts for IL-10, TIGIT, CTLA-4, and LAG3 and was therefore annotated as Treg-like CD8^+^ cells (**Supplementary Figure 5**).

**Figure 6:**
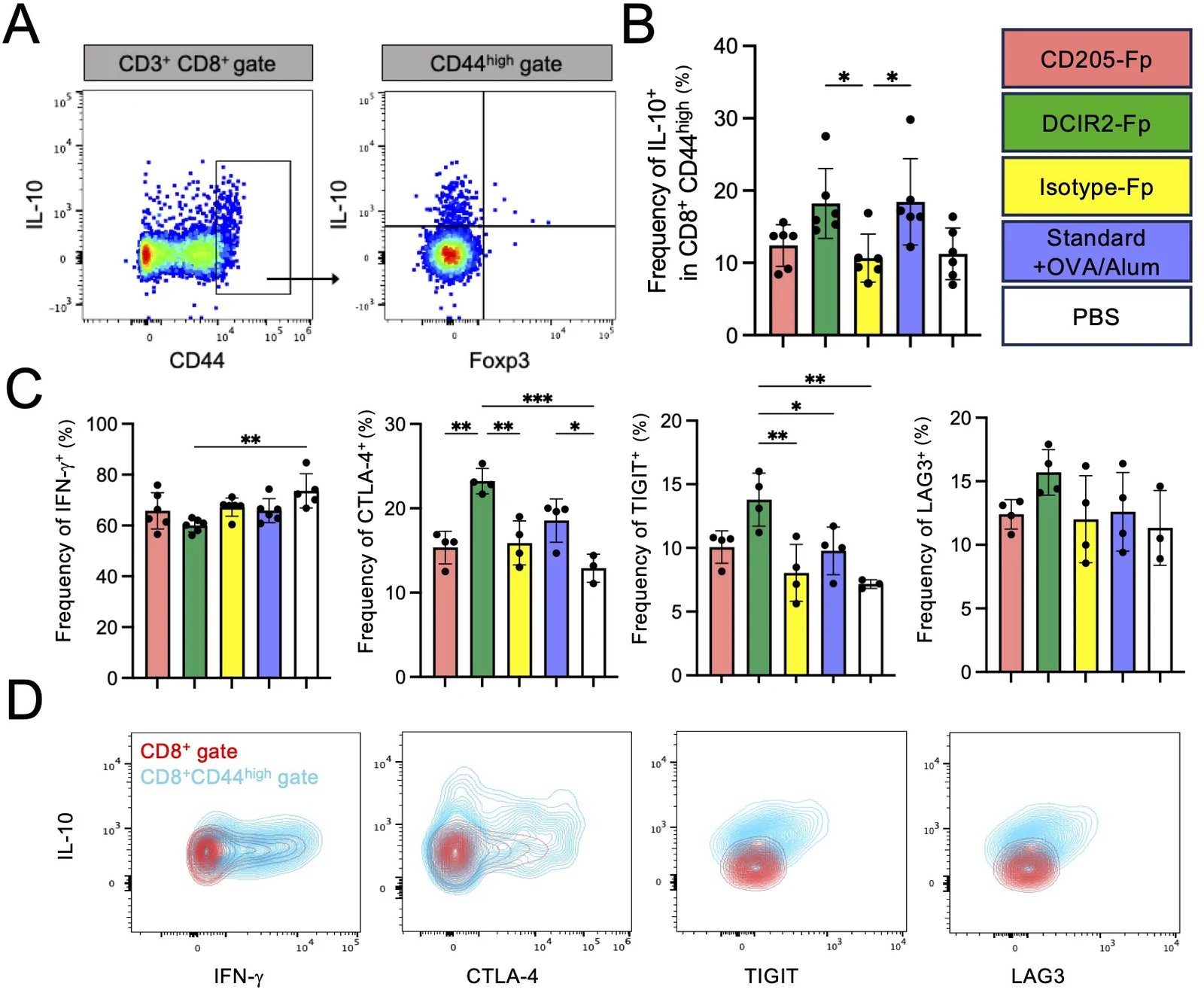
Tolerogenic markers expressed by IL-10-producing CD44^high^ Foxp3^-^ CD8^+^ Treg. Sensitization and desensitization were performed as outlined in **Figure 3A**. On day 80, splenocytes were stimulated with PMA/ionomycin and brefeldin A for 5 h and subsequently analyzed by flow cytometry. **(A)** The gating strategy follows **Suppl. Figure 1** for CD3^+^ and CD8^+^ T cells but includes further gating on CD44^high^ cells to focus on the dominant IL-10-producing cells using the ITIB IL-10 reporter mouse. **(B)** Graphs show the frequencies of IL-10^+^ cells within the CD3^+^ CD8^+^ CD44^high^ gate under the indicated treatment conditions. Data are from *n* = 6 mice per group. **(C)** Graphs show the frequencies of CD8^+^ CD44^high^ T cells expressing the indicated activation and regulatory markers under the indicated treatment conditions. Data are from *n* = 3–6 mice per group. **(D)** Representative flow cytometry plots showing cellular co-expression of IL-10 with the respective markers within the CD8^+^ CD44^high^ gate, overlaid on total CD8^+^ T cells, each from the DCIR2-Fp group. Statistics were performed using one-way ANOVA with Tukey’s multiple comparisons test. ***p < 0.001; **p < 0.01; *p < 0.05.

To gain further insights into the Treg-like CD8^+^ cells, we performed flow cytometric analyses for typical regulatory markers. Within the CD3^+^ CD8^+^ population, the highest IL-10 producers accumulated in a CD44^high^ subset not expressing Foxp3 (**Figure 6A**). The increased frequencies of IL-10 expression under both protective therapies, but not in the control groups, indicate an antigen-specific increase of this presumed regulatory population (**Figure 6B**). The CD44^high^ Treg-like CD8^+^ cells demonstrated significantly increased intracellular CTLA-4 expression and TIGIT on the cell surface. Only trends were observed for higher frequencies of LAG3 expression and lower IFN-γ producers (**Figure 6C**). Among the CD44^high^ CD8^+^ cells, IL-10 producers showed an overlap with the other markers (**Figure 6D**), corroborating the regulatory phenotype.

Together, these data encourage further detailed studies in mice and humans on the role of this newly identified persistent CD44^high^ Foxp3^-^ Treg-like CD8^+^ population in promoting protective IL-10 production, both after long-term Standard therapy combined with OVA/Alum long-term desensitization and even more so following our novel DCIR2-Fp therapy.

## Discussion

In this study, we compared the typical Standard desensitization therapy with a new approach employing antibody fusion proteins targeting different cDC subsets using OVA as an allergen. The Standard desensitization in mice was adopted from clinical treatments using wasp and bee venoms in humans. In this protocol, OVA-sensitized mice are desensitized by a triple treatment with OVA alone (short-term desensitization) followed by two additional treatments using OVA/Alum (long-term desensitization). In our novel approach, fusion proteins consisting of OVA-conjugated antibodies were directed against the cDC1 and cDC2 surface markers CD205 and DCIR2, respectively, thereby enabling selective targeting of these dendritic cell subsets. Both, the Standard therapy and the DCIR2-Fp therapy protected mice against an allergen challenge on day 30 or day 80. On day 30, Standard therapy led to an expansion of IL-10^+^ TIGIT^+^ Foxp3^-^ Tr1, and DCIR2-Fp therapy promoted the development of IL-10^+^ TIGIT^+^ Foxp3^+^ FR4^+^ Treg cells. By day 80, we found that Standard therapy was only protective if additional OVA/Alum injections were performed on days 40 and 60. In contrast, DCIR2-Fp therapy remained fully protective from allergen challenge without further treatment for at least 56 days. Targeting cDC1 with the CD205-Fp or treatment with an Isotype-Fp was not protective. The DCIR2-Fp therapy was characterized by the expansion of a Foxp3^-^ Treg-like CD8^+^ population, which emerged as a dominant IL-10-producing cell type at the long-term time point. These findings suggest that Treg-like CD8^+^ cells are major contributors to protection in this allergy model.

We used a widely distributed and well-investigated OVA-allergy model that follows the principles of sensitization and challenge. In this model, Alum adjuvant is required to sensitize against the otherwise tolerated OVA via MyD88- and uric acid-dependent mechanisms and its potential to polarize T cells towards a Th2 response (47, 48). So-called adjuvant-free mouse models have been described, such as using house dust mites (49). However, it has been debated whether house dust mites may be carriers of microbial compounds working as ‘natural’ or ‘endogenous’ adjuvants (50). Fundamental differences also exist between human and mouse allergy sensitization (51). However, although human allergy is sometimes considered to develop spontaneously, the initial sensitization step also depends on antigenic modifications caused by a large variety of factors, including lifestyle noxes such as tobacco smoke or environmental antigen modifications by diesel particles, heavy metals, or microplastics, just to name a few (52). Thus, all mouse allergy models and human allergic diseases require the antigenic conversion into an allergen that triggers TLRs and other parts of the innate immune system and drives a type 2 immune response (47, 48), similar to what we observed in our model with Alum adjuvant. In any case, it will be interesting to extend the DCIR2-targeting and role of Treg-like CD8^+^ cells to other animal models that come closer to human allergies.

Here, we challenged the mice via i.p. administration of OVA. In contrast, other models perform OVA challenge via the lung to measure airway inflammation and airway hyperreactivity as a model for asthma. Since we were interested in the T cell response after DC targeting and antigen presentation, we found that a challenge via i.p. injection raises a more rapid and systemic anaphylactic response and enabled us to investigate systemic T cell responses such as through scRNA-seq in the spleen.

In our model, we examined CD4^+^ and CD8^+^ bulk T cell responses and the induction of various regulatory T cell subtypes following allergy therapy in C57BL/6 mice. Typically, antigen-specific immune responses in mice are tracked by adoptively transferring TCR-transgenic T cells, which in our model would be the OVA-specific TCR-transgenic CD4^+^ OT-II cells and CD8^+^ OT-I cells. We and others have used the adoptive transfer of these cells for short-term induction of immune responses such as OVA sensitization and short-term conversion of CD4+ T cells into Tr1 or Treg (53–55). However, we previously showed that long-term tolerance induction approaches, such as T-cell anergy or Tr1 cell induction via OT-II cell transfer, were unsuccessful due to nonspecific depletion of the transferred cells (12)(and references therein). Consequently, we could not use OT-II and OT-I cells to track OVA-specific T cell responses over 80 days. This has blurred the analysis of T cell responses in our model, which explains the poor outcome of the detection of CD4^+^ IL-10^+^ Tr1 and Foxp3^+^ Treg by flow cytometry. There are further limitations of the study regarding the Foxp3^+^ Treg analyses. We included technical replicates for the T cell analyses in the spleen to increase the number of data points beyond the actual sample size, i.e., the number of mice. Despite these elevated numbers and data normalization, statistical evaluation by ANOVA did not reveal significant differences. However, a direct comparison of the DCIR2-Fp mice with their control group of Isotype-Fp-treated animals by t-test suggested a low likelihood that DCIR2-Fp animals showed higher frequencies of IL-10^-^ and IL-10^+^ Foxp3^+^ Treg in the spleen. This was not observed when t-tests were applied to the other groups. Together, the poor statistical evidence and the small increase in Foxp3^+^ Treg frequency indicate only a very minor effect. This could be attributed to the above-mentioned fact that only the whole polyclonal T cell repertoire could be analyzed, not selectively antigen-specific T cells. In contrast, our scRNA-seq data pointed to a broader panel of CD4^+^ IL-10^+^ cells in the spleen as suggested by the flow cytometry and previously reported in the literature (56). In addition, the new therapy model allowed the discovery of a CD8^+^ IL-10^+^ T cell population by scRNA-seq, which was confirmed by flow cytometry. As these cells were found to be the dominant IL-10-producing population, this suggests a role also in the IL-10-dependent protection induced by DCIR2-Fp therapy.

To find the most effective dose of fusion protein therapy, three different concentrations were initially tested for desensitization. The low-dose regimen (3/10/30 ng) of anti-DCIR2-Fp showed no effect. In contrast, the high dose protocol (300/1000/3000 ng) and the intermediate dose (30/100/300 ng) showed the best results for desensitization. Previously, lower doses of 1-10 µg were used in immunization protocols, but these were combined with adjuvant treatments (35, 37). The effective intermediate dose of DCIR2-Fp (30/100/300 ng) was 6/10/15 times lower than the soluble OVA dose used in the Standard desensitization protocol (5/10/20 µg). Since the OVA-conjugated DCIR2-Fp has a higher molecular weight, made up of one murine IgG1 (150 kDa) and 2 OVA molecules (each 44 kDa), the amount of pure OVA allergen in the DCIR2-Fp construct is further reduced by approximately 3-fold, to amounts of 10/30/100 ng of OVA. Additionally, the DCIR2-Fp therapy did not require another OVA/Alum regimen on days 40 and 60 to achieve long-term desensitization and full protection on day 80. Therefore, the DCIR2-Fp-therapy provides key advantages over Standard therapy by being effective at much lower allergen doses and requiring less time.

Here, we tested established fusion proteins targeting cDC1 and cDC2 for the treatment of allergy in mice. Both fusion proteins have been successfully employed in models of autoimmunity. CD205 targeting cDC1 has demonstrated protective effects in the EAE model when conjugated with MOG protein (38). In NOD mice, targeting both DC subsets was successful with CD205 conjugates linked to the islet-specific glucose-6-phosphatase catalytic subunit-related protein (39), and cDC2 targeting with the DCIR2 conjugate, which binds a 528–539 fragment of glutamic acid decarboxylase 65, was also protective (57). In these models CD205-Fp reduced the frequency of autoreactive CD8^+^ T cells, and DCIR2-Fp targeting induced tolerogenic CD4^+^ T cells.

The DCIR2 molecule, also named Clec4a4, may be specifically inclined to induce tolerogenic DCs, as it contains a cytoplasmic ITIM motif that transmits immunosuppressive signals (58). However, when OVA-conjugated DCIR2-Fp was combined with an anti-CD40 antibody and a TLR3 agonist, CD4^+^ T cell immunity could be activated in an OVA-melanoma model (37). These findings highlight that inducing antigen-specific tolerance requires the absence of additional immunostimulatory signals. Neither the injection of the CD205-Fp nor the DCIR2-Fp has been previously tested to treat a type-2 immunity model. Our data from the OVA allergy model now show that only cDC2 targeting, not cDC1, is successful in desensitization in allergic mice. Therefore, for both T cell-dependent allergy sensitization and subsequent desensitization, cDC2 seem to play a more prominent role than cDC1.

Since the cDC1/cDC2 dichotomy of DC biology similarly exists in humans, the cDC2 targeting may offer new therapeutic options for human allergies. The question remains which surface marker other than murine DCIR2 could be used in humans to obtain a comparable desensitization success. The murine DCIR2 molecule has no direct human equivalent. While mice have genes for DCIR1-4, humans only have one DCIR gene. Additionally, human DCIR is expressed on all DC subtypes, monocytes, and granulocytes in the blood, making it non-specific for cDC2 (59). Therefore, a new target specific for the cDC2 subtype is needed. One potential candidate is CD301b (macrophage Gal/GalNAc lectin 2, MGL2), which marks cDC2 in both mice and humans and preferentially induces Th2 responses (60, 61) as well as Treg (62). However, this marker is not exclusively expressed by cDC2; it is also found on macrophages and MoDC (63, 64) and seems to be upregulated to represent a functional state rather than a stable lineage marker (61).

In allergy models during the sensitization phase against allergens, cDC2 and monocyte-derived DC (MoDC) subtypes have been shown to transport the allergen to the draining LN (65). At the same time, cDC1 and plasmacytoid DC are thought to act protective (65). In an allergy treatment model, CD205^+^ B7^high^ migratory cDC1 were shown to carry intranasally applied OVA allergen to the draining LN, where their antigen presentation and IL-10 production led to the generation of Tr1 (27). This tolerogenic migratory DC phenotype largely overlaps with steady-state migratory DCs, which appear semi-mature and continuously transport self-antigens from peripheral organs to the draining LN to induce Tr1 or Foxp3^+^ Tregs (24, 32). However, such desensitization using allergen alone shows only limited success, even in human allergy patients. Our data suggest a major role for cDC2 in OVA desensitization, although it is unclear whether these OVA-targeted cells migrate to the lymph node to prime T cells. Furthermore, since, unlike the cDC1 subset, cDC2 do not cross-present antigens to CD8^+^ T cells, it remains enigmatic how the cross-presentation may occur (35, 37, 41, 66, 67). Intraperitoneal OVA injection has been shown to stimulate CD8^+^ T cells exclusively by cDC1 but not cDC2 (68). This dichotomy is likely also true for cross-tolerance induction. Thus, some way of antigen transfer from migratory cDC2 to the LN-resident bystander cDC1 may provide a likely route of the CD8^+^ T cell activation, as we observed already in another model (69). Alternatively, a role of MoDC for antigen transport and/or cross-presentation may be involved (65).

Since we directly targeted cDC2 with the DCIR2-Fp, we expected a subsequent and dominant effect on CD4^+^ T cells. The results obtained from the Standard therapy on day 30 showed IL-10^+^ Tr1 locally in the peritoneum and tracheobronchial LN, while on day 80 Foxp3^+^ Tregs were increased systemically in the spleen. However, they appeared in the IL-10^-^ fraction. The DCIR2-Fp therapy induced no Tr1 but TIGIT^+^ FR4^+^ IL-10^+^ Foxp3^+^ Tregs on day 30 in the spleen. However, it failed to maintain this until day 80. This may point to an expansion of tTreg as indicated by the FR4 marker (44), which are activated due to their IL-10 and TIGIT expression. Since this population declines by day 80, their role in the long-term protection from allergen challenge remains unclear. The timing of T cell measurements and the type of allergy are important factors, as shown in other studies. In a study on allergic rhinitis, a higher frequency of Foxp3^+^ Treg, Tr1, and Breg was observed in human peripheral blood only after 4 years, but not after 2 (70). In another human study, it was shown that the frequency of Foxp3^+^ Treg in peripheral blood decreased one month after starting AIT against wasp venom and returned to baseline after six months (71). In children undergoing AIT who had both respiratory and non-respiratory allergies, the number of Foxp3^+^ Treg increased, whereas in children with only respiratory allergies, the number of Foxp3^+^ Treg decreased (72).

For both successful long-term Standard and DCIR2-Fp therapy, we identified a major transcriptional increase of IL-10 in the CD8^+^ T cells on day 80 by scRNA-seq, which was confirmed by flow cytometry. The frequency of CD8^+^ cells was already increased in the peritoneum on day 30, but the cells appeared IL-10^-^. Thus, their role at this early time point remains unclear. The fact that not only our novel approach but also the Standard approach induces this Treg-like Foxp3^-^ CD8+ T cells raises the question of whether these cells have been overlooked in previous analyses of the OVA model or are specifically induced by our i.p. challenge. This remains unclear and requires further clarification in additional models.

A role for IL-10^+^ CD8^+^ T cells in the control of allergies has been reported before (73), but currently, the mechanism of their induction and maintenance remains unclear, as they also seem to be IL-10 producers during long-term Standard therapy. Suppressive CD8^+^ T cells can be divided into several subsets, which may or may not express Foxp3 and share many immunosuppressive mechanisms, including IL-10 secretion (21). Foxp3^+^ CD8^+^ Treg have been reported to regulate semi-allogeneic Th2 responses in neonatal mice through a partly IL-10-dependent manner (74). In an OVA asthma model in BALB/c mice, a reduction in IL-10^+^ Foxp3^+^ CD8^+^ Treg was observed, and their adoptive transfer improved clinical allergy symptoms (75). The induction of IL-10^+^ Foxp3^-^ CD8^+^ Treg in allergy has not been reported before.

Since direct detection of IL-10 is below the detection limit, we employed PMA/ionomycin restimulation of spleen cells to determine clear IL-10 signals in the scRNA-seq. For the flow cytometry analyses, we also used ITIB reporter mice, which proved superior over intracellular IL-10 antibody staining, ELISA, or Legendplex™ data for IL-10 detection (43). These enabled us to identify a subset of CD44^high^ CD8^+^ T cells as the dominant producers of IL-10. PMA/ionomycin delivers stronger and nonspecific signals as compared to antigen-specific or CD3/CD28 antibody triggering, typically used for T cells. On the other hand, the use of PMA/ionomycin triggers all cell types, including innate immune cells, to recall the cytokines they are primed to secrete. We cannot fully exclude that such strong nonspecific stimulation may also induce OVA-independent IL-10 secretion, particularly by innate immune cells. However, we expect CD8^+^ T cells, as components of the adaptive immune system, to require antigen-specific priming to produce IL-10 after 3 h restimulation with PMA/ionomycin. Notably, an increased CD8^+^ T cell frequency was already observed in the peritoneum on day 30 and all our injections were administered via i.p. route. The peritoneum is enriched for memory T cell phenotypes, including tissue-resident memory cells (T_RM_) (76, 77). Thus, it remains unclear whether a specific subset of IL-10-producing Foxp3^-^ Treg-like CD8^+^ T cells expressing CTLA-4, TIGIT and LAG3 was systemically recruited from the peritoneum. A specific CD8^+^ Treg subset in the peritoneum has not been described so far. Of note, this CD8 induction was only observed in the DCIR-2-Fp-treated group.

DCIR2-Fp therapy suggest a central role for IL-10 in mediating protection in both, short-term and long-term allergen challenges. Anti-IL-10R blockade administered one day before or after short-term DCIR2-Fp desensitization abolished protection, as did treatment one day before the day-80 OVA challenge. This is consistent with previous studies showing that protection following desensitization in the OVA asthma model is abrogated by anti-IL-10R antibody treatment (78). The importance of IL-10 for desensitization against house dust mites has also been demonstrated in mice lacking the IL-10R on CD4^+^ T cells (79). While IL-10, which is also considered a master regulator in human AIT (80), can be produced by multiple cell types, T and B cells are thought to be its main sources during allergy protection (46).

To gain a deeper understanding of the mechanisms behind protection in IL-10-dependent DCIR2-Fp therapy, we performed scRNA-seq on spleen cells, which are a systemic source of immune cells. While canonical markers clearly identified the myeloid cell subsets, the NK cell clusters appeared largely homogeneous. In both clusters, IL-10 production did not seem to contribute to DCIR2-Fp therapy significantly, and myeloid cells even showed lower IL-10 transcription compared to allergic mice that received no therapy. Conversely, B cells and T cells displayed a more diverse range of subsets, suggesting broader functional roles. IL-10 transcripts in well-known IL-10-producing B cells, such as plasma cells or B1 regulatory cells (81), remained fairly consistent across untreated mice and both treated groups. Surprisingly, the largest increase in IL-10 transcripts in T cells induced by DCIR2-Fp treatment was observed in Treg-like CD8^+^ cells, which were Foxp3^-^ (21). Interestingly, a smaller but noticeable increase in IL-10 transcripts was also seen in the long-term Standard therapy group compared to untreated, allergic animals.

Mice that received DCIR2-Fp therapy and a blocking anti-IL-10R antibody on day 79 were not protected from anaphylaxis on day 80. This further supports the widely accepted view that IL-10 plays a crucial role in successful human AIT, lending support to the idea of using IL-10 as a therapeutic agent. However, attempts to apply exogenous IL-10 as a treatment have failed. Children with food allergies expressing allergen-specific IgE showed fewer T cells that can spontaneously release IL-10 without antigen stimulation (82). Since IL-10 is crucial in allergy research, efforts have also been made to administer exogenous IL-10 as a treatment. Yet, this was unsuccessful, indicating that the complex immunomodulatory effects cannot be replicated by exogenous IL-10 (83). Conversely, another study on allergic rhinitis demonstrated that administering recombinant murine IL-10 reduced eosinophils, mast cells, and Th2 cells, but also lowered the number of IL-10-positive cells (84). Thus, further studies are needed to evaluate IL-10 targeting as a therapy for allergic diseases.

In conclusion, our novel DCIR2-Fp therapy targeting cDC2 with OVA provided longer-lasting protection against allergen challenge at lower allergen doses compared with the Standard therapy. This long-term protection was IL-10-dependent. Elevated frequencies of Tr1 or Foxp3^+^ Treg, or their IL-10 production, did not primarily contribute to the protection as predicted. Instead, a subset of Foxp3^-^ Treg-like CD8^+^ cells was identified as the major IL-10-producing cell type. Together, our results encourage further investigation on the role of cDC2 and Treg-like Foxp3^-^ CD8^+^ T cells in allergy desensitization using additional models and in human studies.

## Materials and methods

### Mice

C57BL/6J wild-type mice were initially purchased from Charles River (Sulzfeld, Germany). ITIB mice on a C57BL/6J background [141] were kindly provided by Dr. Frank Thiele (Munich, Germany). All mice were subsequently bred in our institutional animal facility and maintained under specific pathogen-free conditions. Both male and female mice were used. Within each individual experiment, mice were matched for age and sex across groups. All animal experiments were conducted in accordance with the German Animal Welfare Act and were approved and supervised by the local authorities (Regierung von Unterfranken; reference numbers 2–1389 and 500-13/21, allergen immunotherapy).

### Sensitization and desensitization of mice

A sensitization mixture was prepared by combining 10 μg of OVA with 10 μg of Alum, mixing thoroughly by pipetting up and down, and incubating in the dark at room temperature for 45 minutes. After incubation, PBS was added to reach a total volume of 250 µl. To induce sensitization, each mouse received an i.p. injection of 250 µl of the OVA/Alum/PBS mixture on day 0 and a second time on day 10.

Short-term desensitization was performed by i.p. injections of increasing concentrations (5/10/20 µg) of endotoxin-free OVA (Ovalbumin EndoFit™, InvivoGen) in 250 µl PBS on days 20, 22, and 24, respectively. When fusion proteins were used for desensitization, they were similarly administered i.p. in 250 µl PBS on days 20, 22, and 24, using one of three dose regimens: low (3/10/100 ng), intermediate (30/100/300 ng), or high (300/1000/3000 ng). For long-term desensitization, mice received i.p. injections of an OVA/Alum/PBS mixture on days 40 and 60, prepared as described for sensitization.

### Antibodies and fusion proteins for injection

To block IL-10 signaling in mice, 1 mg anti-IL-10R blocking monoclonal IgG1 antibody (clone 1B1.3A, Bio X Cell) or a control IgG1 antibody (clone HRPN, Bio X Cell) was injected i.p. once either one day prior to desensitization (day 19) or one day after completion of desensitization (day 25) in the short-term protocol, or on day 79 in the long-term protocol, as indicated. Allergen challenge was performed one day after antibody administration.

Antibody-antigen fusion proteins were generated by transient transfection of HEK293T cells, as previously described (35, 37, 41). Briefly, plasmids encoding the original variable regions of the two hybridomas, the murine light chains (LC) and murine IgG1 heavy chain (HC), with the IgG1 carrying a mutation (D265A) that affects a glycosylation site to prevent binding to Fc receptors, were introduced via calcium phosphate precipitation. Supernatants were collected after 1 week and concentrated using ammonium sulfate precipitation. Antibodies were purified by affinity chromatography on Protein G Sepharose beads (GE Healthcare). Endotoxins were removed via Triton X-114 phase separation and measured by LAL assays (Lonza or Thermo Fisher) to ensure levels below 0.001 EU/µg. The fusion proteins were controlled by SDS-PAGE with subsequent Coomassie staining or Western blot. Further, all used fusion protein batches were tested for their stimulatory capacity by T cell proliferation assays.

### Allergen challenge and scoring of mice

To assess desensitization efficacy, mice were challenged with a 250 µl i.p. injection of 40 µg OVA in PBS on either day 30 or day 80. Rectal temperature was measured before and 45 minutes after injection. During this period, mice were monitored for behavioral and physiological responses, including scratching, twitching, panting, piloerection, swelling, cyanosis, and use of respiratory muscles, which were scored using a predefined cumulative scoring system. Each parameter was evaluated individually, and higher point values were assigned for prolonged symptom duration. The total anaphylaxis score for each mouse was calculated as the sum of all recorded parameters. All evaluators underwent prior training to ensure consistent application of the scoring criteria. Assessments were performed under blinded conditions, with independent evaluations to ensure consistency and reproducibility across experiments.

### Preparation of single cell suspensions, and restimulation of cells *in vitro*

Peritoneal cells were collected by vigorous peritoneal lavage with PBS as described (85). Spleens and LNs were harvested and processed into single-cell suspensions using standard methods. Briefly, single-cell suspensions were generated by mechanically dissociating lymph node and spleen tissues through a 70-µm cell strainer using a syringe plunger. Lymph node cell suspensions were washed with PBS to maximize cell recovery, while splenic samples underwent sequential red blood cell lysis before lysis was quenched with PBS. Peritoneal, LN and splenic cells were then centrifuged, resuspended in PBS, and viable cells were counted prior to downstream experiments. Single-cell suspensions were seeded *in vitro* in a 24-well plate at a density of 2×10^6^ cells/well in 1 ml Iscove’s Modified Dulbecco’s Medium. Activation was performed using 50 ng/mL phorbol myristate acetate (PMA, Sigma-Aldrich) and 1 µg/mL ionomycin (Sigma-Aldrich) for 5 h. When indicated, brefeldin A (10 µg/ml, Sigma-Aldrich) was added during the last 4 h. Given the expected minimal or undetectable cytokine production under direct *ex vivo* conditions, PMA/ionomycin stimulation was extended for 72 h prior to cytokine detection.

### ITIB substrate staining

IL-10 expression was assessed using IL-10 reporter mice that express β-lactamase under the control of the IL-10 promoter (43). Following restimulation as described in the section above, cells were incubated *in vitro* with 1.3 µM CCF4-AM (Invitrogen) and 3.6 mM probenecid (Sigma-Aldrich) at room temperature for 90 minutes.

### Flow cytometry

The murine, directly conjugated antibodies CD3 – PerCP-Cy5.5 (clone: 145-2C11), CD44 – Brilliant Violet 785 (clone: IM7), CD8 – Brilliant Violet 510 (clone: 53-6.7), CD4 – Alexa Fluor 700 (clone: GK1.5), CD73 – Brilliant Violet 650 (clone: TY/11.8), CD69 – Brilliant Violet 650 (clone: H1.2F3), TIGIT – PE (clone: 1G9), LAG3 – PE-Cy7 (clone: C9B7W), IFN-γ – PE-Cy7 (clone: XMG1.2), CTLA-4 – PE (clone: UC10-4B9) were all purchased from BioLegend. Foxp3 – APC (clone: REA788) was bought from Miltenyi. FR4 – Brilliant Violet 650 (clone: 12A5) was obtained from BD Opti Build. Surface staining was conducted by incubating 10^6^ cells in 50 µl FACS buffer (0.1% BSA in PBS) containing fluorescently conjugated antibodies for 30 minutes at 4 °C. To block nonspecific binding to FcγRII/III, FACS buffer was supplemented with 50% 2.4G2 hybridoma supernatant. Live/dead staining was performed using Viability Dye eFluor 780, followed by an additional 15-minute incubation. Cells were fixed with Fix/Perm buffer (eBioscience) for 20 minutes at room temperature. Intracellular staining was performed by permeabilizing cells and incubating them in 50 μL of Perm buffer (eBioscience) containing fluorescently labeled antibodies for 1 h at room temperature. Following washing with Perm buffer, cells were resuspended in FACS buffer and analyzed on an Attune NxT flow cytometer (Thermo Fisher Scientific). Data analysis was carried out using FlowJo v10 (Tree Star).

### LEGENDplex™ analysis

Cell culture supernatants were collected and stored at -20 °C. The thawed samples were then analyzed for the presence of specific cytokines using a customized LEGENDplex™ Mouse Th Cytokine Panel (BioLegend, San Diego, CA, USA) according to the manufacturer’s protocol. The samples were measured on the Attune NxT, and analyzed by the LEGENDplex™ data analysis software (BioLegend, San Diego, CA, USA).

### Single-cell RNA sequencing

On day 80 after long-term Standard therapy or DCIR2-Fp treatment mice were challenged i.p. for 45 min with OVA. Then, single-cell suspensions derived from mouse spleens were stimulated with PMA (50 ng/mL) and ionomycin (1 µg/mL) for 3 h. Then the cells were cell-sorted into CD45^+^ CD11b^+^ and CD45^+^ CD11b^-^ cells and pooled at a 1:1 ratio to relatively enrich for myeloid cells. Then the cells were frozen until processed using the 10x Genomics Chromium Single Cell 3′ v3.1 platform (10x Genomics) according to the manufacturer’s protocol (CG000315_RevF). To enable multiplexing of multiple samples within a single library preparation, cells were labeled with TotalSeq-A hashtag oligonucleotide (HTO) antibodies (BioLegend). Briefly, individual samples were incubated with sample-specific TotalSeq-A antibodies, thoroughly washed to remove unbound antibodies, and subsequently pooled before undergoing microfluidic partitioning.

The pooled cells were loaded onto the Chromium Controller, where individual cells were encapsulated into Gel Bead Emulsions (GEMs), droplets containing a gel bead with barcoded oligonucleotides. Each oligonucleotide includes a 10x cell barcode, a unique molecular identifier (UMI), and an oligo-dT sequence that captures polyadenylated mRNA molecules. Inside each droplet, cells were lysed, and mRNA molecules were reverse-transcribed to produce barcoded cDNA, ensuring that transcripts from each cell were uniquely labelled. After reverse transcription, emulsions were broken, and the barcoded cDNA was purified, amplified, and used to create both gene expression and hashtag oligonucleotide (HTO) libraries.

Approximately 10,000 cells were targeted for capture per reaction. Libraries were prepared using the Chromium Single Cell 3′ Library and Gel Bead Kit v3 (10x Genomics), following the manufacturer’s recommendations for amplification and indexing. Sequencing was performed on an Illumina NextSeq 2000 system using a P3 flow cell in paired-end (PE) mode with the following read configuration: Read 1: 28 bp (cell barcode and UMI), i7 index: 10 bp, i5 index: 10 bp, Read 2: 90 bp (transcript sequence). Libraries were sequenced to a depth of approximately 50,000 reads per cell for the gene expression libraries and 5,000 reads per cell for the HTO libraries, in accordance with 10x Genomics recommendations.

After assessing sequencing quality using FASTQC, libraries were processed with Cell Ranger 7.2.0, mapping them to the mouse reference GRCm38. Subsequently, demultiplexing was performed separately for each cell type using the HOT Demux function from the Seurat package, with a positive quantile set to 0.85. Cells with fewer than 200 detected genes, more than 10% mitochondrial genes, or over 60% ribosomal genes were considered low quality and removed. Doublets were identified with the DoubletFinder R package and excluded. The data then underwent normalization and scaling, while variable features were identified using the vst method in Seurat. The top 2000 variable genes were used to conduct PCA, followed by 30 PCs for further dimensional reduction. Cell types were assigned using canonical markers. Signatures were calculated with the AddModuleScore function in Seurat. Visualization was accomplished using the SCPubr and scCstomize R packages.

### Statistical analysis

Global one-way ANOVA with was used on each dataset to evaluate overall differences. One-way ANOVA was followed by Tukey’s *post-hoc* test to analyze multiple comparisons. In addition, unpaired, two-tailed t-tests were performed to evaluate the comparisons of each treatment group with its corresponding and closest experimental control (Standard therapy versus PBS group, fusion protein therapy group versus isotype control group). These comparisons were specified *a priori* based on the experimental design and biological hypotheses. The specific statistical test used for each dataset is listed in the corresponding figure legend. Statistical significance is indicated as **** p < 0.0001; *** p < 0.001; ** p < 0.01; * p < 0.05.

## Data Availability

The datasets presented in this study can be found in online repositories. The names of the repository/repositories and accession number(s) can be found below: https://www.ncbi.nlm.nih.gov/geo/, GSE326802.
