# Supplementary material for: Low-dose allergen targeting to cDC2 induces long-term protection associated with induction of regulatory-like IL-10+ Foxp3- CD8+ T cells in mice
Source: Front Immunol. 2026 Aug 25;17:1846923. doi: 10.3389/fimmu.2026.1846923 (PMC13551728; doi:10.3389/fimmu.2026.1846923)
Supplement: Supplementary file 1 [file DataSheet1.pdf]

Suppl. Figure 1

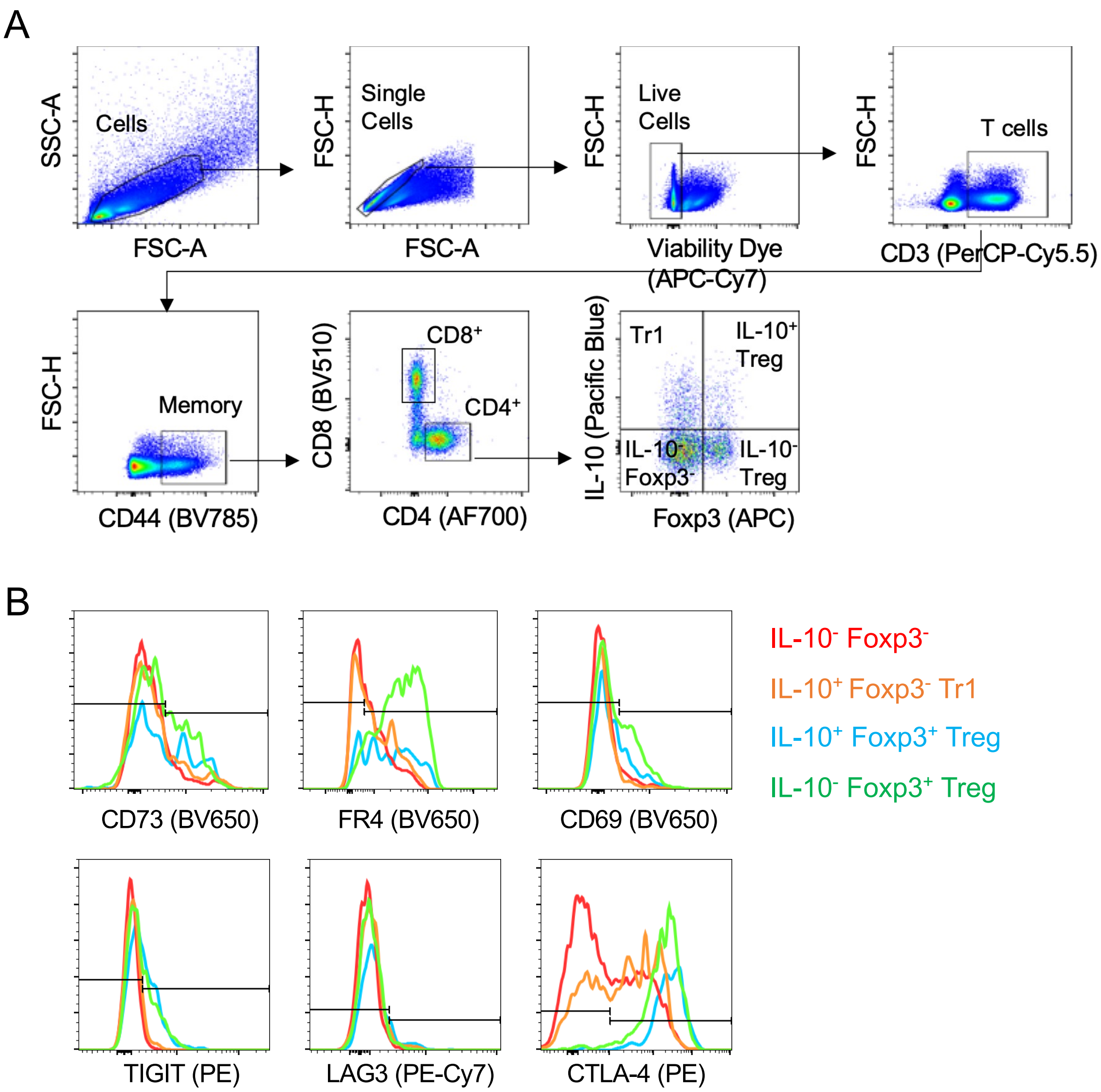

Suppl. Figure 1: Gating strategy for regulatory T cell subsets and example stainings of markers used for further characterization.

Mice were sensitized as outlined in Fig. 1A. On day 11, splenocytes were stimulated with PMA/ionomycin, and brefeldin A for 5 h and subsequently analyzed by flow cytometry. **A.** Representative flow cytometry plots and gating strategy for T cell subsets. The gating strategy included doublet exclusion and restriction to viable cells. IL-10 expression was detected using the ITIB reporter mouse. $CD3^{+}$ $CD4^{+}$ $CD44^{+}$ T cells were further subdivided into IL-10^{+} $Foxp3^{-}$ Tr1 and IL-10^{+} $Foxp3^{+}$ Treg or IL-10^{-} $Foxp3^{+}$. **B.** Representative histograms showing the expression of the indicated tolerance- and activation-associated markers in the respective T cell subsets.

Suppl. Figure 2

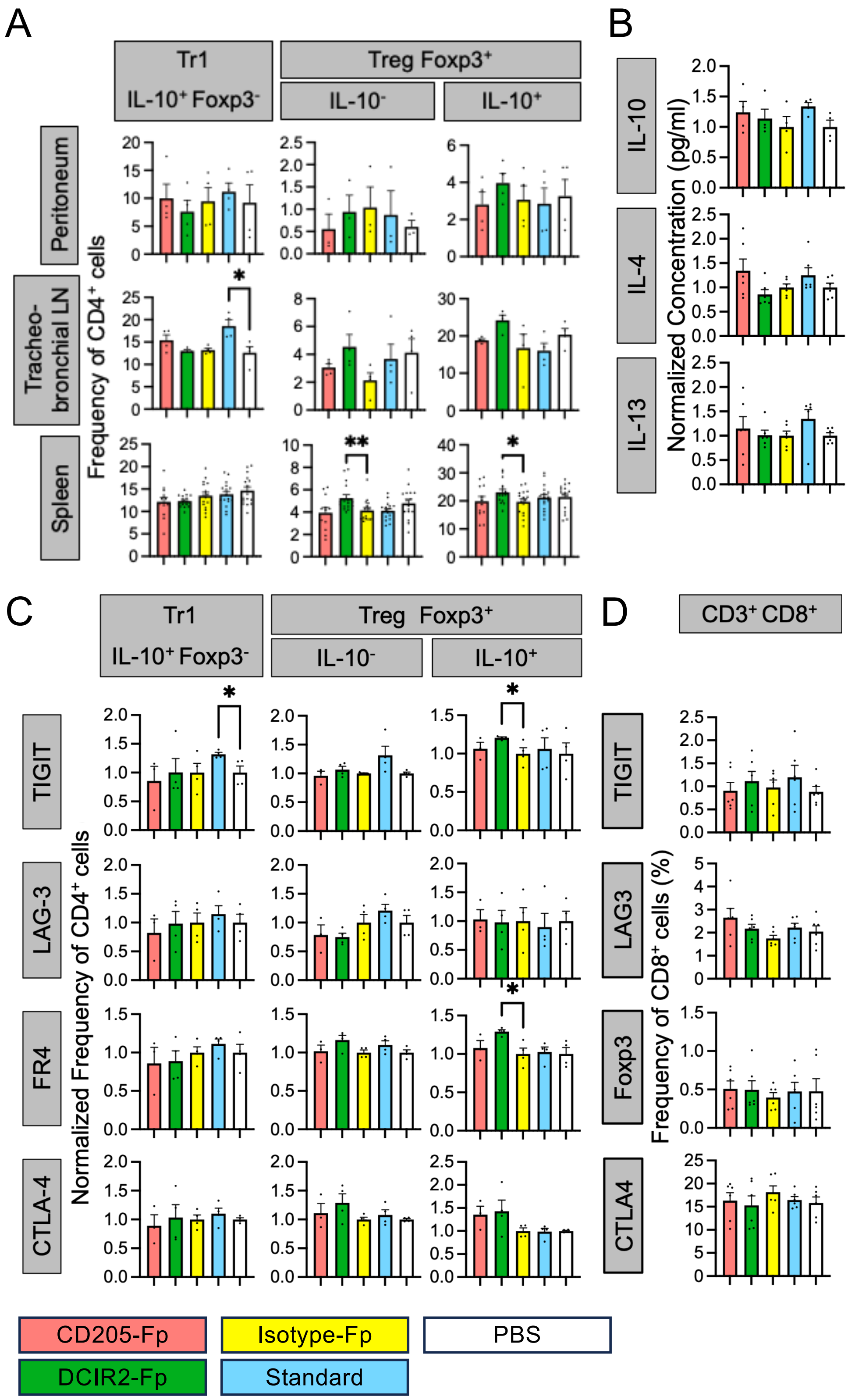

Suppl. Figure 2: Increased expression of TIGIT on Tr1 cells after short-term Standard therapy and increased TIGIT and FR4 expression on IL-10^{+} Tregs after DCIR2-Fp therapy.

Mice were sensitized and desensitized as outlined in Fig. 1A. **A.** On day 30, cells from the peritoneal cavity, tracheobronchial lymph nodes, and spleen were stimulated with PMA/ionomycin, and brefeldin A for 5 h and subsequently analyzed by flow cytometry. Graphs show the non-normalized frequencies of CD4^{+} T cells within the Tr1 and IL-10$^{+/-}$ Treg gates under the indicated treatment conditions, corresponding to the normalized data presented in Fig. 2A. The gating strategy is shown in Suppl. Fig. 1A. Data are from n = 3–6 mice per group (additional technical replicates were included for spleen samples). **B.** On day 30, splenocytes were stimulated with PMA/ionomycin for 72 h. Cytokine concentrations in the culture supernatants were quantified using a LegendPlex^{TM} assay. Graphs show the normalized concentrations of the indicated cytokines under the indicated treatment conditions. Data are from n = 4–6 biological replicates. **C, D.** On day 30, spleen cells were stimulated with PMA/ionomycin, and brefeldin A for 5 h and subsequently analyzed by flow cytometry. Graphs show the normalized frequencies of CD4^{+} T cells within the Tr1 and IL-10$^{+/-}$ Treg gates (C) and of CD8^{+} T cells (D) expressing the indicated tolerance markers under the indicated treatment conditions. Data are from n = 3–4 mice per group. **B, C.** Values from the CD205-Fp and DCIR2-Fp groups were normalized to the corresponding Isotype-Fp control group. Values from the Standard therapy group were normalized to the PBS control group. Normalization was calculated by setting the Isoltype-Fp or PBS group = 1 and relating it to the experimental groups (group x 1 / PBS). Statistical analyses by ANOVA did not appear to be significant. Additional unpaired, two-tailed *t*-tests between groups were applied and are indicated as ** $p < 0.01$; * $p < 0.05$.

Suppl. Figure 3

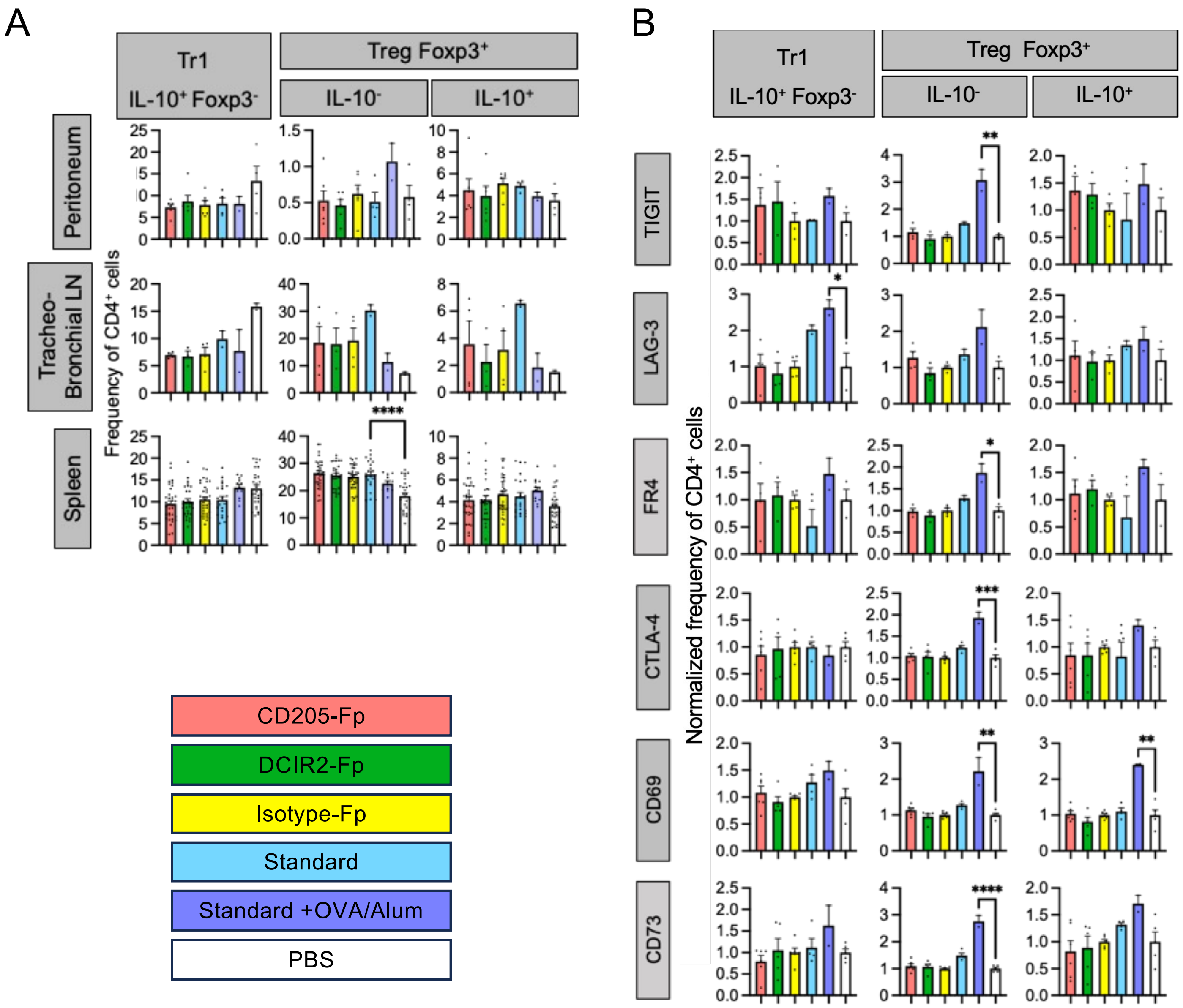

Suppl. Figure 3: Increased expression of tolerance and activation markers mainly on IL-10⁻ Tregs cells after long term Standard therapy, while DCIR2-Fp therapy shows no changes.

Mice were sensitized and desensitized as outlined in Fig. 3A. On day 30, splenocytes were stimulated with PMA/ionomycin, and brefeldin A for 5 h and subsequently analyzed by flow cytometry. The gating strategy is shown in Suppl. Fig. 1A. **A.** Graphs show the non-normalized frequencies of $CD4^+$ T cells within the Tr1 and IL-$10^{+/-}$ Treg gates under the indicated treatment conditions, corresponding to the normalized data presented in Fig. 4A. Data are from n = 3–6 mice per group (additional technical replicates were included for spleen samples). **B.** Graphs show the normalized frequencies of cells within the Tr1 and IL-$10^{+/-}$ Treg gates expressing the indicated tolerance- and activation-associated markers under the indicated treatment conditions. Values from the CD205-Fp and DCIR2-Fp groups were normalized to the corresponding Isotype-Fp control group. Values from the Standard therapy groups were normalized to the PBS control group. Normalization was calculated by setting the PBS group = 1 and relating it to the experimental groups (group x 1 / PBS). Data are from n = 3–4 biological replicates. Statistical analyses by ANOVA did not appear to be significant. Additional unpaired, two-tailed t-tests between groups were applied and are indicated as **** $p < 0.0001$; *** $p < 0.001$; ** $p < 0.01$; * $p < 0.05$.

Suppl. Figure 4

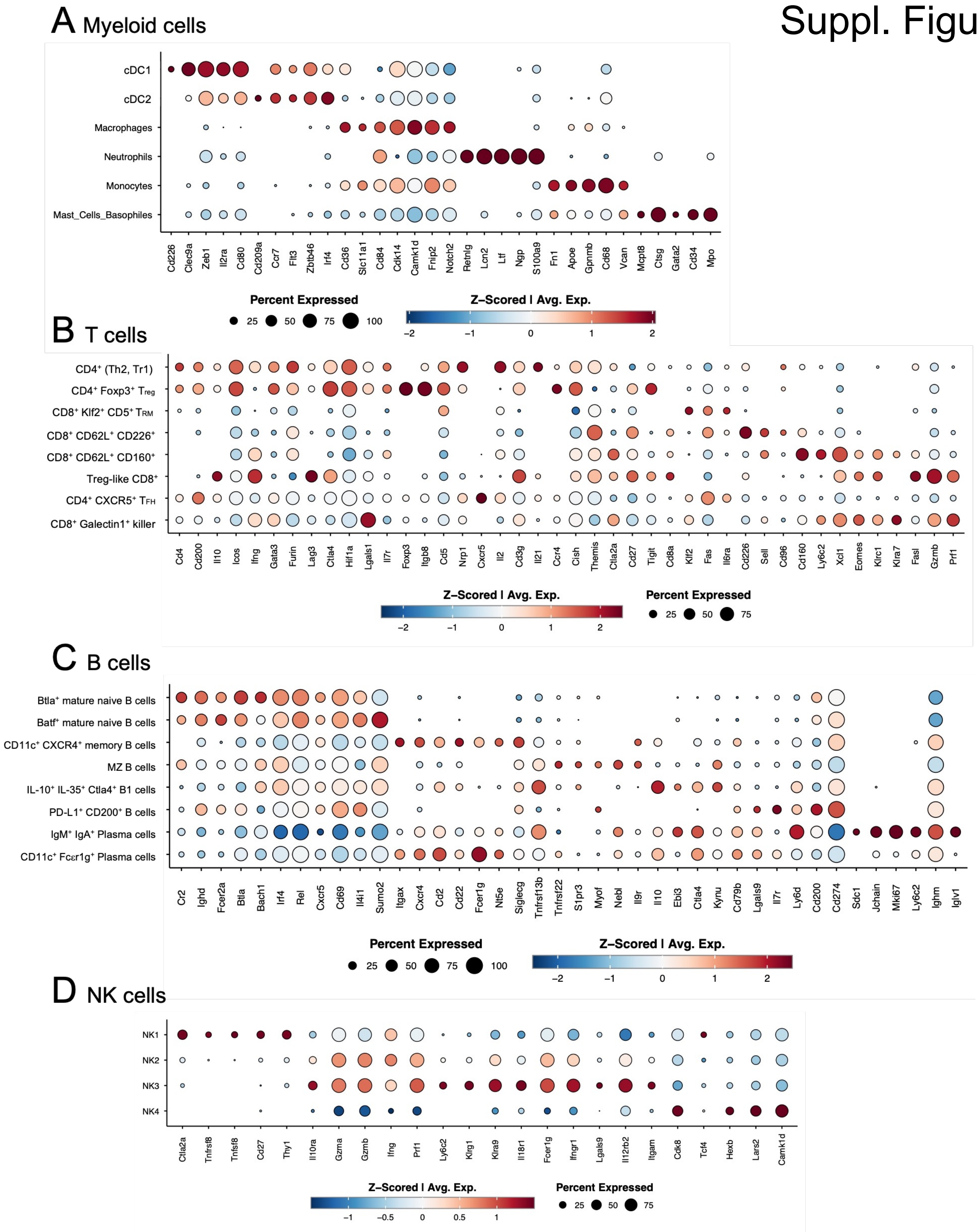

Suppl. Figure 4: Annotation of scRNA-seq clustered immune cell types.

Mice were sensitized and desensitized as outlined in Fig. 3A. On day 80, mice were challenged with 40µg OVA in PBS. Then splenocytes were stimulated with PMA/ionomycin and brefeldin A for 3 h. Spleen cells of all groups were collected and enriched for otherwise scarce myeloid cells by sorting CD45⁺ CD11b⁺ cells and then pooling them with the total spleen cells at a 1:1 ratio. Then the pooled cells were subjected to scRNA-seq. **A–D.** Dot plots showing the Z-scores of the average expression of genes used for cell type annotation. Data were pooled from mice receiving no therapy, long-term Standard therapy, or DCIR2-Fp therapy. Dot size indicates the percentage of cells expressing the indicated gene, and color indicates the scaled average expression (Z-score).

Suppl. Figure 5

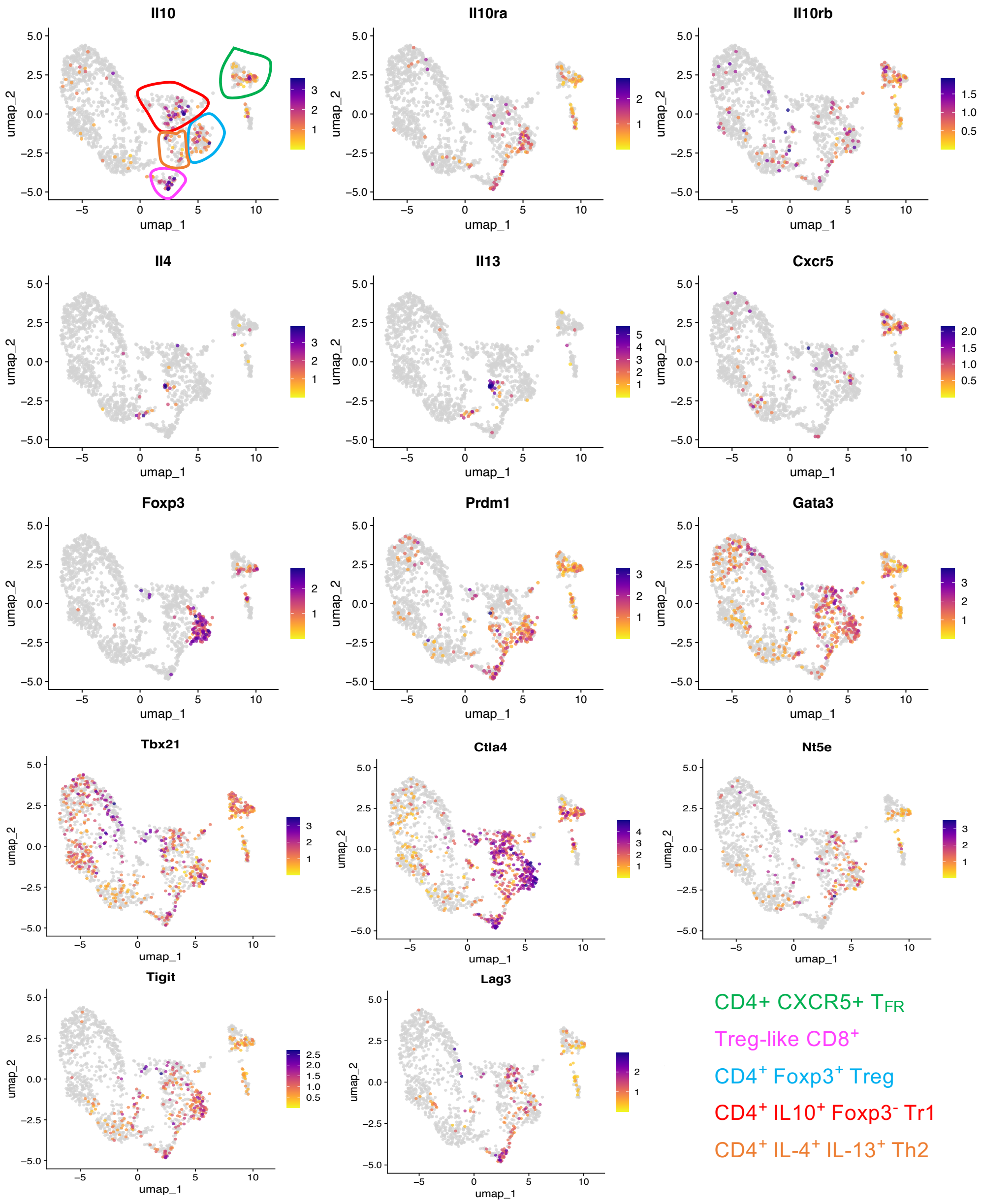

Suppl. Figure 5: UMAP plots from by scRNA-seq identifiy the major IL-10-producing T cell subsets from d80 spleen cells.

Mice were sensitized and desensitized as outlined in Fig. 3A. On day 80, mice were challenged with 40µg OVA in PBS. Then splenocytes were stimulated with PMA/ionomycin and brefeldin A for 3 h. Spleen cells of all groups were collected and enriched for otherwise scarce myeloid cells by sorting CD45+ CD11b+ cells and then pooling them with the total spleen cells at a 1:1 ratio. Then the pooled cells were subjected to scRNA-seq. UMAP plots showing the normalized expression of the indicated genes in T cell populations. Major IL-10-producing cells are highlighted by color-coded circles indicating the annotated T cell subsets. Data were pooled from allergic mice receiving no therapy, long-term Standard therapy, or DCIR2-Fp therapy.
